# Excited‐State Proton Transfer in Push‐Pull N‐Methyl Pyridium Reversible Super‐Photoacids Ruled by Intramolecular Hydrogen‐Bond‐Like Interactions

**DOI:** 10.1002/chem.202502289

**Published:** 2025-11-10

**Authors:** Alessio Cesaretti, Carmela Bonaccorso, Martina Alebardi, Alessandro Grasso, Rossana Quinzi, Benedetta Carlotti, Fausto Elisei, Cosimo G. Fortuna, Anna Spalletti

**Affiliations:** ^1^ Department of Chemistry Biology and Biotechnology and Center of Excellence on Innovative Nanostructured Materials (CEMIN) University of Perugia Via Elce di Sotto 8 Perugia 06123 Italy; ^2^ Department of Chemical Sciences University of Catania Viale Andrea Doria 6 Catania 95125 Italy

**Keywords:** intramolecular charge transfer, photocatalysis, super‐photoacidity, ultrafast spectroscopy

## Abstract

Three push‐pull N‐methyl pyridium phenols have been thoroughly investigated for their photoacid behavior through stationary and time‐resolved spectroscopic techniques. Fluorimetric titrations revealed a clear excited‐state proton transfer (ESPT) with negative pKa* values for the two molecules with the phenol attached in ortho relative to the double bond, so as to be regarded as super‐photoacids. Conversely, no proton transfer was observed when the hydroxyl group was in the para position. Quantum‐mechanical calculations confirmed that intramolecular hydrogen‐bond‐like interactions, only attainable in the ortho isomers, are strengthened by the charge transfer process occurring upon excitation, resulting in enhanced acidity. Ultrafast transient absorption and fluorescence up‐conversion (UC) spectroscopies allowed direct observation of the ESPT process on the picosecond time scale, in agreement with the negative pKa*. In addition, nanosecond laser flash photolysis unraveled the recombination of the conjugated base with the proton once returned to the ground state. The time for this recombination was slowed down by one order of magnitude in the presence of a proton scavenger. These findings demonstrated both the reversible nature of these super‐photoacids, thus regulating the pH of the nearby environment through light irradiation, and their ability to give a proton to a second molecule, for potential photocatalytic applications.

## Graphical abstract

Push‐pull A^+^‐π‐D N‐methyl pyridinium derivatives were found to act as efficient reversible superphotoacids. Upon excitation, they undergo a fast ESPT but recombine with the released proton when the excitation is turned off. In the ortho isomers, intramolecular hydrogen‐bond‐like interactions between the hydroxyl group and the ethenic hydrogen are strengthened in the excited state, as a consequence of ICT, leading to enhanced photoacidity.

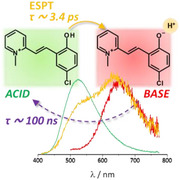

## 1 Introduction

Photoacids are molecular species whose excited state is more acidic than their ground state by several orders of magnitude in terms of acid dissociation constants (Ka). When the excited‐state pKa (pKa*) becomes negative, photoacids can be referred to as super‐photoacids because of their efficient and fast deprotonation upon light irradiation.^[^ ^1^, ^2^, ^3^, ^4^, ^5^ ^]^ This is based on the possibility of transferring a proton either to a second partner, commonly the solvent, by excited‐state proton transfer (ESPT), or intramolecularly, through excited‐state intramolecular proton transfer (ESIPT).^[^ ^6^, ^7^ ^]^ Both processes become feasible when the conjugated excited base is stabilized by virtue of the redistribution and delocalization of the negative charge formed upon deprotonation.^[^ ^8^, ^9^, ^10^ ^]^ Intramolecular charge transfer (ICT) processes can therefore grant this stabilization, making push‐pull compounds potential candidates as photoacids.^[^ ^11^ ^]^ The ESPT phenomenon is particularly common in aqueous solution, thanks to the tendency of water to form hydrogen bonds and accept the transferred proton from the photoacids;^[^ ^2^ ^]^ however, ESPT has also been observed in water/organic solvent mixtures and pure organic solvents, including nonprotic ones, provided that the strength of the photoacid is adequate.^[^ ^12^ ^]^ The rate of the ESPT process also depends on the strength of the photoacids, which can be classified in four regimes depending on their pKa* values.^[^ ^13^, ^14^, ^15^ ^]^ Regime I consists of those photoacids with a positive pKa* for which the ESPT happens in tens of picoseconds and becomes progressively slower, up to the nanosecond time scale, as the pKa* value increases. Regime II applies when the pKa* varies from − 4 to 0; in this case, the ESPT gets faster and competitive with solvation dynamics. In Regime III, pKa* ranges between − 6 and − 4, the ESPT is controlled by solvation and has the same time as the average solvation time. Photoacids with pKa* <−6 belong to Regime IV. They are the strongest photoacids, and they are typically characterized by the presence of electron‐withdrawing groups, which greatly enhance the acidity of the proton in the excited state. Among these, there is a cyanine fluorochrome dye (QCy7, pKa*~ −6.1);^[^ ^16^, ^17^ ^]^ 8‐aminopyrene‐1,3,6trisulfonic acid (APTS, pKa*~ −7);^[^ ^18^ ^]^ and the strongest photoacid known to date, phenol‐carboxyether dipicolinium cyanine (QCy9, pKa* = −8.5).^[^ ^5^, ^17^ ^]^ For these photoacids, the ESPT to the solvent happens in 100 fs both in water and other protic solvents like alcohols, being limited by the vibrations between the OH group of the acid and the oxygen atom of the solvent receiving the released proton. The fast ESPT expected for super‐photoacids makes ultrafast time‐resolved spectroscopy an invaluable tool for temporally following the evolution of the photoacid excited‐state dynamics and proving the deprotonation of the excited molecule upon ESPT.^[^ ^19^, ^20^, ^21^, ^22^ ^]^

Photoacids can be grouped into photoacid generators and reversible photoacids.^[^ ^23^, ^24^, ^25^ ^]^ The former are molecules that, once deprotonated, undergo a chemical reaction that does not allow the conjugated base to reform the initial acid; the latter are instead capable of recombining with a proton when the irradiation is turned off and go back to their protonated form, which is thermodynamically favored in the ground state. Hence, reversible photoacids attract much interest, as they can promote light‐controlled pH jumps.^[^ ^26^, ^27^, ^28^, ^29^, ^30^ ^]^

This stimulus‐responsive behavior can be exploited for a number of applications, ranging from locally influencing the pH at a molecular level in a biological environment to catalyzing chemical reactions or controlling dynamic processes.^[^ ^23^, ^31^, ^32^, ^33^ ^]^ For example, photoacids have been widely used for the optical control of enzymatic activity;^[^ ^34^ ^]^ further, merocyanine‐based photoacids have been employed as catalysts in photoacid‐mediated ring‐opening polymerization reactions driven by visible light^[^ ^35^ ^]^ or else super‐photoacids have recently been exploited to trigger the protonation of a surfactant, causing the self‐propulsion of the oil droplets formed by the surfactant molecules.^[^ ^36^ ^]^ Understanding the mechanisms of photoacidity is thus of the utmost importance for guiding the synthesis of novel compounds that can fulfill specific tasks.

**Scheme 1:**
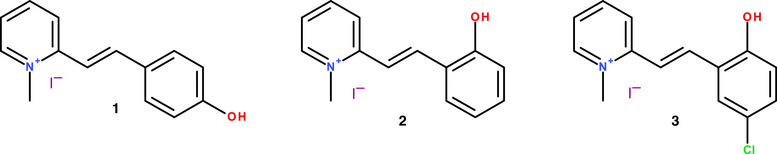
Structures of the investigated compounds reported as iodide salts.

In this work, three push‐pull N‐methyl pyridium molecules have been synthesized and studied for their ground‐state acidochromism, that is, the dependence of their spectral properties on the medium acidity,^[^ ^37^, ^38^, ^39^, ^40^ ^]^ and their photoacid behavior (Scheme 1).

Some hints on the photoacidity of this class of molecules have already been given.^[^ ^41^, ^42^, ^43^, ^44^, ^45^ ^]^ In fact, compound **1** has already been scrutinized, and, as anticipated by the Förster cycle, its pKa of 8.57 is expected to undergo a significant drop in the excited state, that is, pKa* = 1.85.^[^ ^41^ ^]^ However, this value is not enough for **1** to be considered a super‐photoacid. Therefore, with the aim being to enhance the photoacidity of the molecule, its ortho isomer (compound **2**) and a chlorinated derivative (compound **3**) were prepared and herein deeply investigated, as well. Spectrophotometric and spectrofluorimetric titrations were carried out to determine the pKa and possibly pKa* values of the three compounds. Femtosecond‐transient absorption and fluorescence up‐conversion (UC) measurements were performed to follow in time the plausible involvement of the ESPT phenomenon, while nanosecond laser flash photolysis was employed to draw a complete picture of their photoinduced dynamics. Finally, with the aid of TD‐DFT calculations, the role of charge distribution in regulating the photoacid behavior of the three N‐methyl pyridinium derivatives was assessed.

## 2 Results and Discussion

### Synthesis

Compounds **1–3** (Scheme 1) were synthesized via base‐catalyzed Knoevenagel condensation between the 1,2‐dimethylpyridinium ion and the proper aldehyde; following work‐up procedures and crystallization, the compounds were isolated in their cationic form as iodide salts (see Scheme S1 in Supplementary Material). The compounds are readily soluble in water, both at near‐neutral pH and in acidic conditions; only compound **3** showed slightly lower solubility in neutral solution when the main species in solution is the neutral zwitterion. Compounds **1–3** were obtained as pure trans isomers, as evidenced by the ethylenic protons' J coupling constants (≈16.0 Hz) in the NMR spectra (Figures S1–S12).

### Spectrophotometric and Fluorimetric Titrations

**Figure 1:**
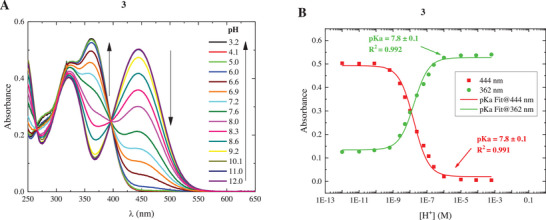
Spectrophotometric titration (panel A) of compound **3** in a large pH interval and relative pKa fitting (panel B) at 362 nm (green curve) and 444 nm (red curve), corresponding to the maxima of the protonated and deprotonated forms, respectively. Arrows point to changes in the absorption spectrum as the pH decreases.

Compounds **1–3** were first characterized for their ground‐state acidity by means of spectrophotometric titrations conducted from pH 12 to pH 2. All three molecules exhibited acidochromism, with their solutions being yellow under alkaline conditions while bleaching as the acidity in the sample was increased (Figures 1A and S13). In fact, an absorption band centered between 430 and 444 nm was peculiar to the basic forms of the three compounds, with compounds **2** and **3** also having a distinguished UV absorption feature around 325 nm. As the pH decreased, these bands became progressively less intense, while another band appeared at about 360 nm being characteristic of the acidic form. For compound **1**, the absorption of the base was greater than that of the acid, as opposed to compounds **2** and **3**, for which the acid's UV absorption was higher. Each titration allowed the identification of a definite isosbestic point, which serves as an indicator of the acid‐base equilibrium between two differently‐protonated species: the zwitterionic form, deprotonated at the hydroxyl group, and the protonated cation. The processing of the data (Figures 1B and S14) gave the values of the ground‐state pKa, which was found to be around 8.5 for compounds **1** and **2**, in line with literature reports,^[^ ^41^ ^]^ and reduced to 7.8 for compound **3**. The addition of the chlorine atom, thanks to its electron‐withdrawing ability, is responsible for the greater acidity of the latter compound by impoverishing the electron density around the oxygen of the hydroxyl group and making it easier for the H^+^ proton to be released in the solution.

**Figure 2:**
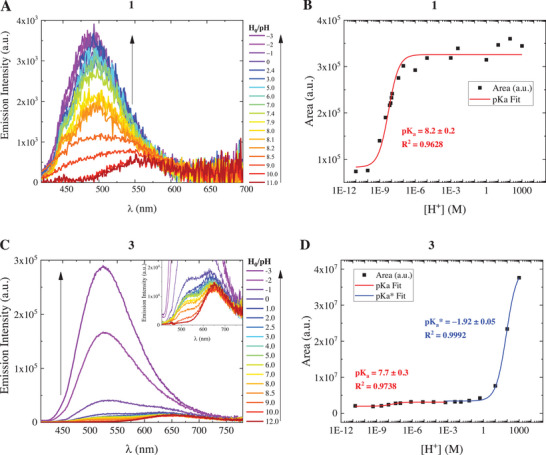
Spectrofluorimetric titrations of compounds **1** (panel A) and **3** (panel C) in a large pH/H_0_ interval. Inset in panel C: zoom‐in at low emission intensities to appreciate the changes in the emission between pH 12 and 6. Arrows point to changes in the emission spectrum as the pH/H_0_ decreases. Relative pKa/pKa* fitting for compounds **1** (panel B) and **3** (panel D) obtained by plotting the areas subtended by the emission spectra as a function of [H^+^].

Subsequently, spectrofluorimetric titrations were performed in the 2–12 pH range and also exploring negative H_0_ values, by exciting the samples at the wavelength of the isosbestic point (Figures 2 and S15). Starting from pH 12, a weak fluorescence centered at 560 nm for compound **1** and 640–650 nm for compounds **2** and **3** was recorded. As for compound **1** (Figure 2A), its emission underwent a monotonic enhancement together with a marked blue shift by increasing the acidity of the solution up to pH ≈ 6, and then it remained nearly constant upon further lowering the pH. The plotting of the fluorimetric data (Figure 2B) revealed a sigmoidal curve that could be fitted to obtain a value for the pKa of 8.2 ± 0.2, which matches the one retrieved from the absorption titration. This suggests that the emission of the excited states reflects the acid‐base equilibrium reached in the ground state, with the protonated compound **1** weakly emitting at 560 nm under alkaline conditions and its deprotonated cationic form exhibiting higher fluorescence with a maximum at 485 nm in neutral and acidic solutions. The prevalent form in the ground state, once brought to the excited state, is the one responsible for the emission, so that the pKa measured is that of the ground state. A peculiar and different behavior was instead found for both compounds **2** and **3** (Figures 2C and S15); their red emission remained clearly detectable when decreasing pH to low values, down to about 1. Concurrently, a green fluorescence centered at 505 and 524 nm for **2** and **3**, respectively, grew as soon as the pH decreased below 11/10, but it markedly turned on only at negative H_0_ values. By plotting the areas subtended by the spectra as a function of [H^+^], a double trend was observed (Figures 2D and S15). The first jump can be fitted to obtain a pKa value closely comparable to the one given by the spectrophotometric titrations (although uncertainties are somewhat larger due to low fluorescence emission peculiar to these molecules, especially in their deprotonated forms), while the other inflection point revealed a second and negative pKa (−1.4 and − 1.9 for **2** and **3**, respectively). The latter can thus be attributed to the excited‐state pKa (pKa*) for the ESPT leading to the deprotonation of the two molecules. In fact, the absorption spectra ensured that under pH 6, the only species present in the ground state is the protonated one; however, the fluorescence spectra unveiled that, despite exciting exclusively the protonated cation, both the higher‐energy emission of the protonated form and the long‐wavelength emission of the deprotonated one are recorded up to pH 0, implying that a partial acid‐base re‐equilibration happens in the excited state between pH 0 and 6. Exclusively at negative H_0_, the emission detected is purely that of the excited acid species. The negative values measured for the pKa* pointed out a strong photoacidity for the two molecules, which is also confirmed by the theoretical pKa* value determined by applying the Förster cycle as outlined in the Experimental Section in the Supplementary Information. This method, based on thermodynamic considerations by excluding entropic factors, allows the pKa* to be estimated from the spectral properties of the two species involved in the acid‐base equilibrium (Figures S16–S18 and Table S1).^[^ ^46^ ^]^ The negative pKa* values obtained by the Förster cycle are in perfect agreement with the experimental ones for compounds **2** and **3**, while a value of 2.0 was predicted for compound **1**. This value, which is about 4 orders of magnitude higher than those of the other two molecules, suggests that, despite its excited state being much more acidic than its ground state (pKa = 8.5 vs. pKa* = 2.0), this is not enough for **1** to be a factual photoacid. These different behaviors imply that the attachment position of the phenolic ring plays a pivotal role in ruling the ESPT process. Table 1 summarizes the pKa and pKa* values determined for the acid/base equilibrium of compounds **1–3**.

**Table 1:** Acid dissociation constants in the ground and excited states (expressed as pKa and pKa* values, respectively) for the acid/base equilibrium of compounds **1–3**.

	pKa		pKa*	
Compound	Abs^[^ ^a^ ^]^	Fluo^[^ ^b^ ^]^	Fluo^[^ ^b^ ^]^	Theor^[^ ^c^ ^]^
**1**	8.5 ± 0.1	8.2 ± 0.2	‐	2.0
**2**	8.4 ± 0.1	9.0 ± 0.2	−1.4 ± 0.2	−1.4
**3**	7.8 ± 0.1	7.7 ± 0.3	−1.92 ± 0.05	−1.9

^[a]^ Determined by spectrophotometric titrations analyzed by best fitting of absorbance recorded at suitable absorption wavelengths.

^[b]^ Determined by spectrofluorometric titrations analyzed by best fitting of total areas subtended by emission spectra.

^[c]^ Calculated through the Förster cycle according to the equation reported in the Experimental Section in the Supplementary Information.

### Quantum‐Mechanical Calculations

**Figure 3:**
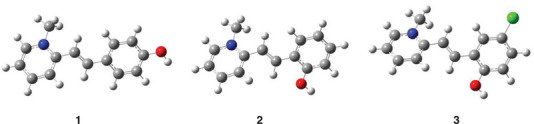
Optimized structures of cationic/protonated compounds **1–3** obtained by wB97XD/6–311 + G(2d,p) @ S_0_ in water.

To support the hypothesis about the importance of the attachment position of the phenolic ring in ruling the ESPT process, quantum‐mechanical calculations were performed aiming at optimizing the molecular geometries in the ground state and visualizing the charge distribution changes upon excitation. Speaking of the possible conformers of the three trans isomers of the investigated molecules, the most stable are the ones with the methyl‐pyridinium oriented on the same side as the ethylenic hydrogen, as this configuration minimizes their steric interaction.^[^ ^47^, ^48^ ^]^ As for the rotation around the single bond between the ethylenic bridge and the phenolic ring, compounds **2** and **3** can exist as two different conformers depending on the orientation of the hydroxyl group relative to the double bond, while **1,** featuring the OH in the para position, is insensitive to this rotation. Both possible conformers for the two ortho‐substituted molecules can give intramolecular hydrogen‐bond‐like interactions between the hydroxyl group and the ethylenic hydrogens.^[^ ^49^ ^]^ The geometry optimization was carried out on the conformer with the OH oriented on the opposite side of the double bond relative to methyl‐pyridinium, giving the structure reported in Figures 3 and S19 for the protonated and deprotonated forms in the ground state, respectively.

**Figure 4:**
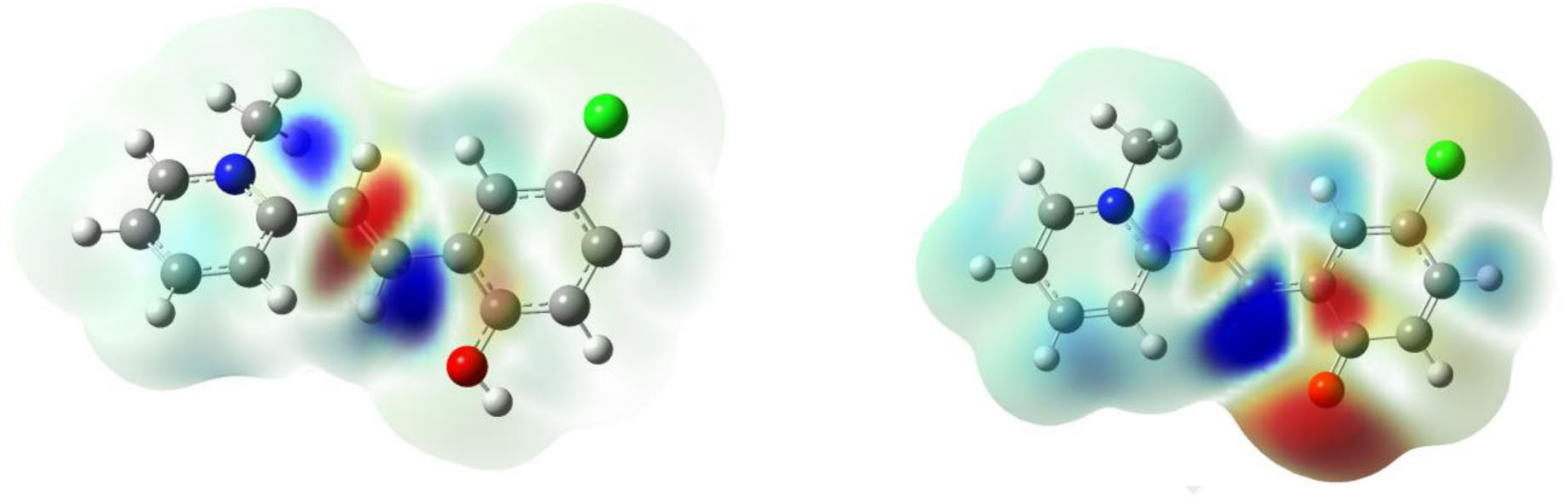
Effect of the S_0_→S_1_ transition on the electron density of 3 in its cationic (left) and zwitterionic (right) forms; increase and decrease of electron densities are represented by blue (+0.0001) and red (−0.0001), respectively.

The first allowed absorption transition is always the S_0_→S_1_, which is mainly described by a π‐π transition from the HOMO to the LUMO orbitals (Tables S4–S8 and Figures S21, S22, S24, S25, S27, S28, S30, S31, S33, and S35). The changes in electron densities revealed that a certain charge transfer from the phenolic ring to the methyl‐pyridinium occurs upon excitation and becomes particularly important in the deprotonated molecules, where the phenolate is a better electron donor relative to the phenol (Figures 4 and S22, S25, S28, and S31). Interestingly, in all cases, a significant increase in electron density is calculated close to the ethylenic hydrogen on the side of the phenolic ring. This means that when the hydroxyl group is present at the ortho position, the intramolecular hydrogen‐bond‐like interaction between the phenolic oxygen and the ethylenic hydrogen is likely to be strengthened. Geometries were also optimized in the excited state (Figure S20), and the effect on the electron density was evaluated on the S_1_→S_0_ transition, as well. The electron density maps for the emission transition show the opposite behavior relative to that in absorption, with a decrease in the region of the ethylenic hydrogen (Figures S23, S26, S29, S32, S34, and S36). This implies that the relevance of hydrogen‐bond‐like interactions is likely to diminish when the molecule returns to its ground state. This interaction, attainable in the excited state of **2** and **3**, makes the phenol more acidic and favors the ESPT, with the consequent release of the proton to the solvent. This peculiar interaction is not possible in **1**, where the hydroxyl group is instead located at the para position. As a consequence, the excited state of **1** is not acidic enough to give a fast ESPT. Tables S2 and S3 summarize bond lengths and angles for the optimized geometry in both the ground and excited states. These data support the hypothesis of the involvement of hydrogen‐bond‐like interactions in regulating the pKa* of the molecular systems under investigation. In particular, when considering the distance between the ethylenic hydrogen and the atom at the ortho position relative to the double bond (which is a hydrogen for **1** and an oxygen for **2** and **3**), values of 2.33, 2.35, and 2.37 Å are predicted for the optimized ground state geometry of the cationic/protonated forms of **1**, **2**, and **3**, respectively. Once promoted to the excited state, the optimized geometries reveal no changes for **1**, while this value is reduced to 2.29 Å for both **2** and **3**. This finding serves as further proof of the occurrence of intramolecular hydrogen‐bond‐like interactions, responsible for the increased excited‐state acidity of **2** and **3**.

### Ultrafast Spectroscopy

**Figure 5:**
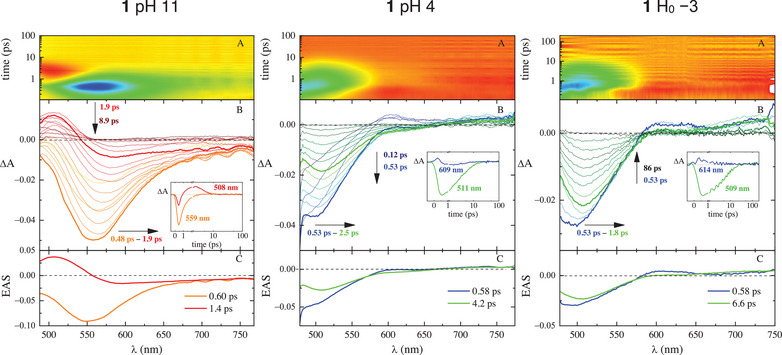
Femtosecond‐transient absorption (fs‐TA) measurements of compound **1** in buffered water at pH 11 (left), pH 4 (middle), and H_0_ = −3 (right) (*λ*_exc_ = 400 nm): panel A, experimental 3D matrix reporting color‐coded ΔA as a function of wavelength and time (ΔA > 0 red, ΔA < 0 green‐blue); panel B, representative spectra at different delay times and representative kinetics (inset) at different wavelengths; panel C, EAS (evolution‐associated spectra) obtained by Global Analysis.

**Figure 6:**
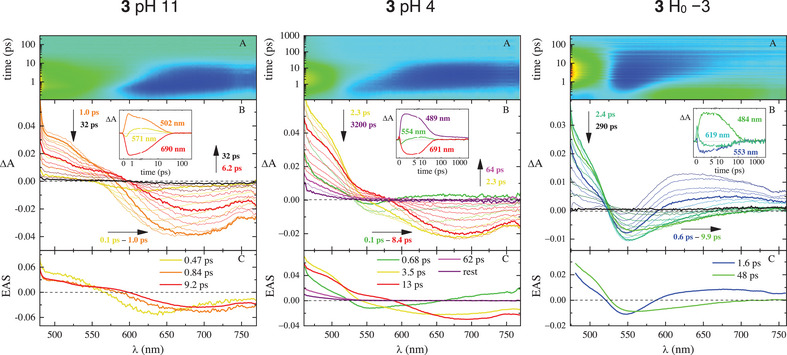
Femtosecond‐transient absorption (fs‐TA) measurements of compound **3** in buffered water at pH 11 (left), pH 4 (middle), and H_0_ = −3 (right) (*λ*_exc_ = 400 nm): panel A, experimental 3D matrix reporting color‐coded ΔA as a function of wavelength and time (ΔA > 0 green‐yellow, ΔA < 0 blue); panel B, representative spectra at different delay times and representative kinetics (inset) at different wavelengths; panel C, EAS obtained by Global Analysis.

With the aim being to follow the ESPT process in time, femtosecond‐resolved transient absorption (TA) and fluorescence UC measurements were performed by exciting the samples at 400 nm under limit pH conditions (pH = 11 > pKa and H_0_ = −3 < pKa* to follow the excited‐state dynamics of the base and acid, respectively). An intermediate pH value of 4 (pKa* < pH < pKa) was instead chosen to prove the ESPT of 2 and 3. The results of the TA experiments are reported in Table 2. The TA measurements carried out at pH 11 (Figures 5, 6, and S30, left graphs) are dominated for all of the three molecules by negative signals due to stimulated emission (SE) in the same region of the stationary fluorescence; plus, a positive signal attributed to excited‐state absorption (ESA) can be observed in the blue side of the explored spectral window. The SE band undergoes a marked red shift in the first picoseconds after the excitation, particularly for 2 and 3, and then it quickly decays in a few picoseconds. The combination of red shift and fast decay points to significant excited‐state stabilization, probably accompanied by ICT, in agreement with the large Stokes shifts and low fluorescence quantum yields (see below) and as anticipated by quantum mechanical calculations. The global analysis of the data revealed three components for 2 and 3 and only two in the case of 1. The ultrafast components of 2 and 3 (*τ* < 1 ps) can be attributed to solvation dynamics: the fastest (*τ* = 0.33 ps for 2 and *τ* = 0.47 ps for 3) to inertial solvation (Solv._i_), overestimated because of the time resolution of the instrumentation, and the other (*τ* = 0.85 ps for 2 and *τ* = 0.84 ps for 3) to diffusive solvation (Solv._d_). The third and longest component (*τ* = 5.6 ps for 2 and *τ* = 9.2 ps for 3) with its SE peaking above 600 nm is assigned to the deactivation of the relaxed ICT state. In the case of 1, the decay is even faster so that only one solvation component can be observed (*τ* = 0.60 ps), followed by a fast decay of the relaxed S_1_ state, whose SE is centered at 585 nm in line with the stationary fluorescence, in just 1.4 ps. Moving to H_0_ = −3 (Figures 5, 6, and S37, right graphs), an SE signal is revealed at shorter wavelengths (around 500 nm for 1 and 550 nm for 2 and 3) relative to what was observed at pH 11, in agreement with the higher‐energy steady‐state fluorescence of the acidic form of the three molecules. A red shift of the SE band is peculiar to the first picoseconds after the laser excitation, then the signal is progressively reduced, and the deactivation becomes complete in tens/hundreds of picoseconds. The global analysis of the data matrix unveiled always two transients: the fastest component characterized by a time of 0.6–1.6 ps is assigned to Solv._d_, likely mixed with some vibrational cooling (VC) contribution, and the longest, with a time of 4.2, 15, and 48 ps for 1, 2, and 3, respectively, to the deactivation of the relaxed S_1_ state. The longer times indicate a reduced stabilization, supposedly due to a reduced ICT character, for the acidic form relative to the basic one. In fact, the deprotonated phenolic ring is a better electron donor than its protonated counterpart. The TA measurements performed at pH 4 proved revealing about the ESPT phenomenon (Figures 5, 6, and S37, middle graphs). Under these pH conditions, the only species present in the solution is the protonated acid for all three investigated molecules. As for 1, the excited‐state deactivation parallels the one detected at negative H_0_ for the acidic form, meaning that no ESPT happens upon excitation, thus corroborating the results of the fluorimetric titration. When it comes to 2 and 3, a peculiar spectral evolution was instead recorded. Right after the laser excitation, a negative SE band is formed around 550 nm, in the same spectral region where the acid emits at H_0_ = −3. Later, in a few picoseconds, this signal undergoes a marked redshift up to 670–690 nm, matching the SE wavelengths of the deprotonated basic form. This finding is a distinct sign of an excited‐state acid‐base re‐equilibration: starting from the ground state where only the acidic form is present, the emission of the protonated cation is first observed, then it quickly evolves toward the region of the base as a result of deprotonation through ESPT. Finally, the base returns to the ground state in tens of picoseconds. The global analysis required five transients to describe the excited‐state evolution of both 2 and 3 at pH 4: the first, with a lifetime shorter than 1 ps and a SE band peaked around 550 nm, represents the pure protonated acidic form as observed at H_0_ = −3; the second transient, with τ = 2.6 and 3.5 ps for 2 and 3, respectively, features a broad band of SE, covering the emission region of both the protonated and deprotonated forms, and is assigned to the ESPT phenomenon; the third and most red‐shifted transient has a spectral profile very much resembling that of the relaxed base at pH 11 (red spectra in Figures 5, 6, and S37, left graphs) and a lifetime of 10 and 13 ps for 2 and 3, respectively. Hence, it can confidently be attributed to the deprotonated molecules. These assignments, summarized in Table 2, have also been validated by the fluorescence UC experiments reported below. The last two transients are characterized only by a small signal of positive ΔA at the blue edge of the spectrum, and their spectral shapes are almost identical to each other: one has a lifetime of tens of picoseconds, while the other exceeds the 3200‐ps temporal window of the experiments.

**Table 2:** Femtosecond‐transient absorption (fs‐TA) results of compounds **1–3** in buffered water at pH 11, pH 4, and H_0_ −3 obtained by Global Analysis and their assignment.

	pH 11		pH 4		H_0_ −3	
Compound	τ /ps	Assignm.	τ /ps	Assignm.	τ /ps	Assignm.
**1**	0.60	Solv.	0.58	Solv.	0.58	Solv.
			4.2	S1 ACID	6.6	S_1_ ACID
	1.4	S_1_ BASE				
**2**	0.33	Solv._i_			1.2	Solv.
	0.85	Solv._d_	0.83	Solv.**/** S_1_ ACID	15	S_1_ ACID
			2.6	ESPT		
	5.6	S_1_ BASE	10	S_1_ BASE		
			53	S_0, hot_ BASE		
			rest	S_0_ BASE		
**3**	0.47	Solv._i_			1.6	Solv.
	0.84	Solv._d_	0.68	Solv./S_1_ ACID	48	S_1_ ACID
			3.5	ESPT		
	9.2	S_1_ BASE	13	S_1_ BASE		
			62	S_0, hot_ BASE		
			rest	S_0_ BASE		

In order to further investigate these transients, the same measurement for 3 at pH 4 was carried out, resorting to a CaF_2_ crystal able to generate white light up to 420 nm (Figure S38). This experiment allowed the spectral profile of the longest transient to be clearly outlined. It features an all‐positive band with a maximum at around 450 nm, nicely overlapping with the absorption band of the deprotonated molecule. This finding offers tangible proof of the ESPT process: in fact, once the protonated cation is brought into the excited state, it undergoes deprotonation, transforming into the zwitterion, which later returns to the ground state. This species, which is not present in the absence of light under these pH conditions, is thus responsible for the TA at long delays. However, it is expected to go back to its acidic form, as the latter is thermodynamically favored in the ground state at pH 4 (pH < pKa). Hence, to prove the reversible nature of the photoacid, nanosecond flash‐photolysis analyses were performed as described below.

In addition, starting from the fluorescent quantum yields measured as described in the Experimental Section in the Supplementary Information (Φ_F_) and the fluorescence lifetimes of the relaxed S_1_ state obtained from the analysis of the TA data (*τ*_S1_), the values of the rate constants for the various processes involved in the deactivation of compounds **1–3** can be found (Tables 3 and S10). Broadly speaking, Φ_F_ are extremely low with values below 0.1% under alkaline conditions and mildly increasing as the pH is reduced. This behavior suggests that nonradiative deactivation pathways, supposedly trans‐cis isomerization and internal conversion, are favored over fluorescence. The further reduction of the emission probability upon deprotonation may come as a result of the increased push‐pull character of the zwitterions, which typically boosts internal conversion. Preliminary tests on the isomerization demonstrated that this process is negligible for the deprotonated molecules, while it plays a role for the acidic forms. By taking this into account, one can carry out an appraisal of the rate constants of the various processes at different pHs. At pH = 11, the only nonradiative pathway is internal conversion; therefore, k_nr_ = k_IC_ = (1 − Φ_F_)/τ_S1_ ∼ 10^11^ s^−1^ for all compounds. At H_0_ = −3, isomerization gains importance and k_nr_ = k_IC_ + k_ISO_ = (1 − Φ_F_)/τ_S1._ This value is 1.5 × 10^11^ s^−1^ for 1; while it is reduced to 6.7 and 2.0 × 10^10^ s^−1^ for 2 and 3, respectively. As for the k_F_ for the emission transition, given the small Φ_F_ and short τ_S1_, values of 10^8^ s^−1^ can always be measured, pointing to fairly allowed transitions in all cases. As a rule, the protonated forms exhibit a higher k_F_ of about 2–3 × 10^8^ s^−1^; this number is slightly affected by deprotonation for 1, while it becomes one‐third for 2 and 3, lowering the probability of the emission. Thus, a twisted ICT state could be likely hypothesized for the deprotonated form of the two ortho derivatives. Interestingly, the k_F_ measured at pH 4 for 1 is comparable to the one obtained for the protonated molecule at H_0_ = −3, implying that the emitting species is the same, as opposed to 2 and 3, for which the k_F_ value at pH 4 resembles that of the deprotonated compound, again confirming that the ESPT is active under these pH conditions. Moreover, by considering the Φ_F_ of the differently protonated molecules under the two limit conditions (H_0_ − 3 and pH 11), the efficiency of the ESPT process can be derived from the Φ_F_ measured at pH 4: in doing so, extremely high ESPT efficiencies of roughly 0.88 and 0.98 can be assessed for 2 and 3, respectively. Hence, k_ESPT_ = Φ_ESPT_/τ_ESPT_ ≈ 3 × 10^11^ s^−1^ can be estimated for both compounds. ESPT is therefore competitive with isomerization and favored over it, being k_ESPT_ (≈ 3 × 10^11^ s^−1^) one order of magnitude higher than that of isomerization k_iso_ (≤ 10^10^ s^−1^).

**Table 3:** Fluorescence properties of compounds **1–3** in buffered water at H_0_ −3, pH 4, and pH 11: fluorescence quantum yields (Φ_F_), lifetimes of S_1_ as obtained by femtosecond TA (*τ*_S1_), fluorescence constants (k_F_ = Φ_F_/*τ*_S1_).

Compound	pH/H_0_	Φ_F_ /%	τ_S1_ / ps	k_F_ /s^−1^
**1^+^**	−3	0.14	6.6	2.1 × 10^8^
**1^+^**	4	0.13	4.2	3.1 × 10^8^
**1**	11	0.030	1.4	2.1 × 10^8^
**2^+^**	−3	0.50	15	3.3 × 10^8^
**2^+^**	4	0.11	10	1.1 × 10^8^
**2**	11	0.056	5.6	1.0 × 10^8^
**3^+^**	−3	1.3	48	2.7 × 10^8^
**3^+^**	4	0.088	13	0.68 × 10^8^
**3**	11	0.065	9.2	0.71 × 10^8^

**Figure 7:**
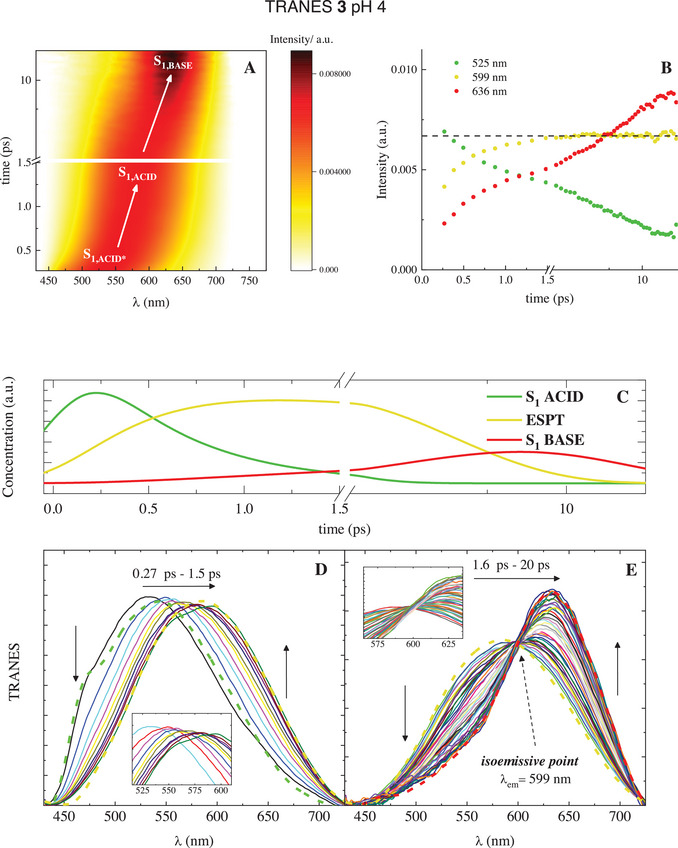
TRANES analysis of femtosecond fluorescence UC data of compound **3** in buffered water at pH 4 (*λ*_exc_ = 400 nm): panel A, 3D matrix reporting color‐coded TRANES intensity as a function of wavelength and time; panel B, intensity variations of TRANES as a function of time at significant wavelengths; panel C, concentration profiles for the transient species detected by Global Analysis; panels D and E, TRANES evolution over time calculated in proper delay time intervals together with the EAS obtained by Global Analysis (dashed lines) reported as limit spectra.

Fluorescence UC measurements were carried out to follow the evolution of the emission spectrum in time and again confirm the ESPT of 2 and 3 (Figures S39–S41). All the measurements show a fluorescence spectrum that shifts toward the red as the delay time from the laser excitation increases, primarily as an effect of the excited‐state stabilization granted by solvation and VC. The global analysis of the data gave the same number of transients with closely equivalent lifetimes as those obtained from the TA experiments, except for the fastest ultrafast component attributed to solvation, which is not always detected in the fluorescence UC measurements because of the reduced temporal resolution of this technique (Table S11). In the case of 2 and 3 at pH 4, a specific data analysis, that is the computation of TRANES (time‐resolved area‐normalized emission spectra), was carried out to gain evidence of the occurrence of the ESPT process (Figures 7 and S42). TRANES, being area‐normalized spectra, are independent of the decrease in fluorescence intensities in time and allow the changes in the spectral shapes to be followed, thus distinguishing common relaxation phenomena from evolutions between different species, like in ESPT.^[^ ^50^, ^51^ ^]^ In particular, relaxation phenomena are visualized as a continuous redshift of the TRANES as a result of progressive energy stabilization, without experiencing a significant change in the spectral profile. Conversely, when there is a passage from one species to another, the emission spectrum changes markedly and this evolution is highlighted by a clear isoemissive point in the TRANES. This perfectly applies to the time‐resolved spectra of 2 and 3 at pH 4: in the first picoseconds after the photoexcitation, when the concentration of the first transient detected decays in favor of the second (Figures 7 and S42, panel C), a continuous bathochromic shift is observed without altering the symmetric bell‐shaped spectrum (Figures 7 and S42, panel D), thus corroborating the assignment of the first lifetime (*τ*_1_ = 0.49 and 0.51 ps for 2 and 3, respectively) to Solv._d_. At longer delays (above 1.5 ps and up to 20 ps) the concentration profiles reveal that the second transient is converted into the third (Figures 7 and S42, panel C). In this time interval, the time‐resolved emission spectra change markedly, with a further redshift of the maximum above 600 nm, a reduction of the full width at half maximum, and the appearance of an asymmetric blue tail (Figures 7 and S42, panel E). These spectral modifications lead to the detection of an isoemissive point, confirming the ESPT between the acidic form and the deprotonated one, happening with the time of a few picoseconds associated with the second transient (*τ*_2_ = 3.2 and 3.4 ps for 2 and 3, respectively). This finding is in line with the classification of photoacids in four different regimes based on their pKa* values, given that the lower the pKa* is, the more efficient and faster the ESPT process gets.^[^ ^13^ ^]^ Regime I is characterized by a positive pKa*, like 1. Photoacids belonging to this regime have medium/weak strength; their ESPT in water happens with a time ranging from tens of picoseconds to the nanosecond time scale as the pKa* moves away from the lower limit value of 0. That is the reason why the photoacid behavior of 1 is not manifest, inasmuch as the ESPT would take place with a time that exceeds the short excited‐state lifetime of the molecule. Regime II consists of those photoacids featuring negative pKa* between − 4 and 0, as is the case with 2 and 3. Their ESPT occurs in less than 20 ps, still being rather slower or at most fairly close to the solvation dynamics depending on the specific pKa*. Thus, a value of about 3 ps for 2 and 3 correlates well with their pKa* of − 1.4 and − 1.9, respectively.

### Nanosecond Transient Absorption: Reversible Photoacidity

**Figure 8:**
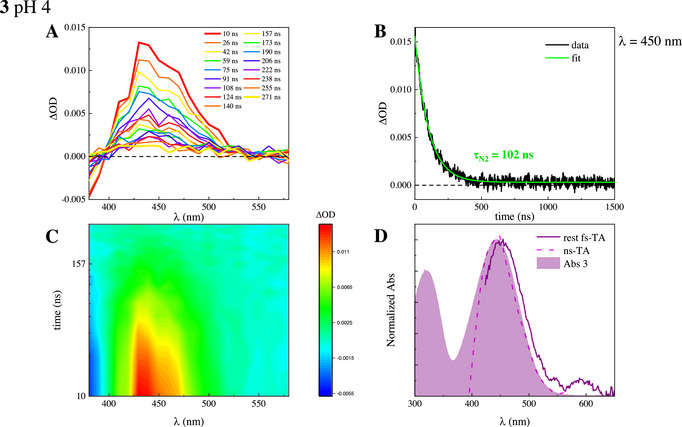
Nanosecond laser flash photolysis measurements of compound **3** in buffered water at pH 4 (*λ*_exc_ = 355 nm): panel A, representative spectra at different delay times; panel B, kinetic recorded at the maximum wavelength of the transient band (*λ* = 450 nm) and its fit in deaerated solution; panel C, experimental 3D matrix reporting color‐coded ΔOD as a function of wavelength and time (ΔOD > 0 yellow‐red, ΔOD < 0 blue); panel D, comparison between spectral shapes of rest absorption in fs‐TA experiment and ns‐TA signal, together with steady‐state absorption spectrum of deprotonated/zwitterionic 3.

With the aim of verifying the reversible nature of the two photoacids, 2 and 3, nanosecond laser flash photolysis experiments were carried out at pH 4 by exciting the solutions at 355 nm and probing the transients in the 380–580 nm range (Figure 8, panels A and C). A negative signal due to GSB was detected below 400 nm, while a positive band centered around 450 nm was revealed as a result of TA. This signal was monitored in time, and it decayed in hundreds of nanoseconds for both compounds (no TA was instead detected for 1 on the same timescale, Figure S43). The kinetics at 450 nm were fitted with a monoexponential function, which gave a lifetime of 108 and 112 ns for 2 and 3, respectively (Figures S44, panel A, and S45). The effect of molecular oxygen was also considered by purging nitrogen in the solutions to remove it, but no appreciable changes in the transient lifetimes were measured (Figures 8, panel B, and S44, panel B). This result allowed the involvement of long‐living excited states, as triplet states, which would instead be greatly affected by oxygen, to be ruled out and corroborated the assignment of the transient to the deprotonated molecule in the ground state. Moreover, its spectral profile overlaps with both the absorption spectrum of the zwitterion and the rest absorption in the femtosecond TA experiment, again confirming its straightforward assignment (Figure 8, panel D). Further, the disappearance of this species in about one hundred nanoseconds proves the reversibility of the photoacid, which returns to the thermodynamically favored protonated form in the ground state once the excitation is turned off.

The photoacidity of these molecules can thus be controlled by light and could potentially be exploited to photocatalyze a reaction. To prove the ability of the investigated photoacids to provide a proton to a second partner, experiments were conducted in the presence of a proton scavenger, that is, acetate at pH 6. Under these pH conditions, acetic acid is in its deprotonated form, while 3 is mostly protonated and can act as a photoacid. The same nanosecond flash‐photolysis measurement was performed, and the typical TA associated with the deprotonated molecules was revealed (Figure S46). Interestingly, in the presence of acetate, protons were kept from recombining with the phenolate of 3, and the time for the disappearance of the deprotonated molecules was enhanced by one order of magnitude, becoming 1.48 µs.

**Scheme 2:**
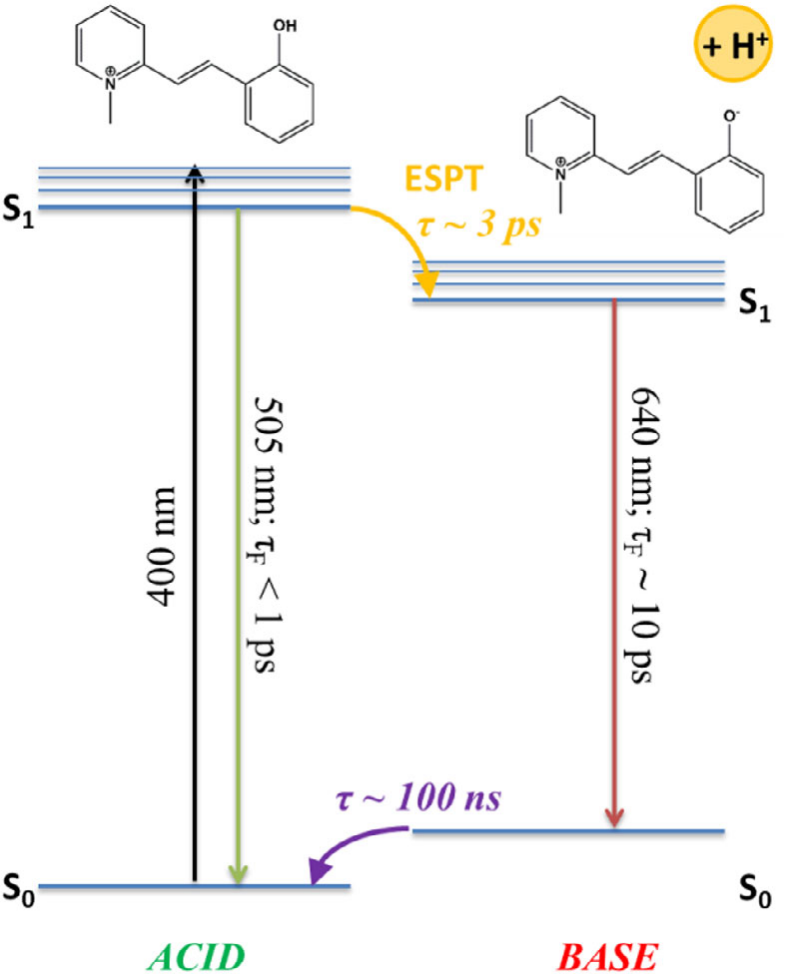
Schematic representation of the deactivation pathways followed by 2 after the excitation of its acid form at pH 4 (pKa* < pH < pKa) and relative time constants. The same scheme would apply to 3.

By gathering the information coming from all the experiments performed, a scheme describing the deactivation pathways of the two photoacids (2 and 3) at pKa* < pH < pKa can be sketched (Scheme 2). Under these pH conditions, the acidic form of the molecule is largely prevalent in the ground state. Once brought to the excited state, it initially shows the typical green emission of the acid, but it undergoes a fast and efficient ESPT process (*τ*_ESPT_ ≈ 3 ps) to form the excited base, whose red‐shifted emission (*λ* ≈ 650 nm) can clearly be revealed. The conjugated excited base returns to its ground state with a time constant of about 10 ps, because its pronounced push‐pull character, by virtue of the enhanced electron acceptor strength of the deprotonated phenol, strongly favors internal conversion over fluorescence. Later, the base in the ground state is back converted to the thermodynamically favored acid with a time of about 100 ns to complete the cycle.

## 3 Conclusion

To sum up, three push‐pull A^+^‐π‐D N‐methyl pyridinium derivatives, where the donor portion is an ionizable phenol, were expressly synthesized and thoroughly scrutinized for their photoacidity. Interestingly, switching the hydroxyl group from the para to the ortho position relative to the double bond resulted in a significant enhancement of the photoacid behavior of the investigated N‐methyl pyridium molecules and allowed clear signs of ESPT to be recognized for both compounds **2** and **3**, as opposed to compound **1**. Spectrofluorimetric titrations in a wide range of pH/H_0_ values in water granted the direct observation of the excited‐state acid‐base re‐equilibration exclusively for the two molecules with the hydroxyl group in the ortho position, with negative values of pKa*. Femtosecond‐resolved spectroscopies (namely, TA and fluorescence UC) played a key role in identifying and following the ESPT process in time, which happens in about 3 ps for both compounds, as is typical of super‐photoacids. In addition to this, TD‐DFT calculations proved fundamental in rationalizing the specific behaviors of the three molecules by relating them to their different charge distributions both in their ground and excited states, unveiling the special function served by intramolecular hydrogen‐bond‐like interactions and charge transfer processes in regulating their photoacidity. The identification of a nanosecond‐living transient, further investigated by nanosecond laser flash photolysis, uncovered the reversible nature of the photoacids, which return to their thermodynamically favored acidic form once the excitation is turned off. They were also found capable of donating a proton to acetate, used as a proton scavenger in light of possible applications as photocatalysts. Hence, compounds **2** and **3** can be regarded as reversible super‐photoacids, and their ability to undergo a fast deprotonation in the excited state might be effectively exploited to locally change and control the pH in a solution by irradiation.

## Supporting Information

The authors have cited additional references within the Supporting Information.^[^ ^52^, ^53^, ^54^, ^55^, ^56^, ^57^, ^58^, ^59^, ^60^, ^61^, ^62^, ^63^, ^64^, ^65^, ^66^ ^]^

## Conflict of Interest

The authors declare no conflict of interest.

## Supporting information



Supporting Information

## Data Availability

The data that support the findings of this study are available in the supplementary material of this article.
