# Supplementary material for: Excited‐State Proton Transfer in Push‐Pull N‐Methyl Pyridium Reversible Super‐Photoacids Ruled by Intramolecular Hydrogen‐Bond‐Like Interactions
Source: Chemistry. 2025 Nov 10;31(70):e02289. doi: 10.1002/chem.202502289 (PMC12712759; doi:10.1002/chem.202502289)
Supplement: Supplementary file 1 — Supporting Information [file CHEM-31-e02289-s001.docx]

**Supplementary Information**

**Excited-State Proton Transfer in Push-Pull N-Methyl Pyridium Reversible Super-Photoacids Ruled by Intramolecular Hydrogen-Bond-Like Interactions.**

*Alessio Cesaretti*^[a]^, Carmela Bonaccorso*^[b]^, Martina Alebardi^[a]^, Alessandro Grasso^[b]^, Rossana Quinzi^[a]^, Benedetta Carlotti^[a]^, Fausto Elisei^[a]^, Cosimo G. Fortuna^[b]^, Anna Spalletti^[a]^*

**Table of contents**

**Experimental Section**

*General* 2

*General Synthetic Procedure* 2

**Scheme S1.** Synthesis of compounds **1-3** 2

*Absorption and Emission Measurements* 3

*Förster cycle for determining pKa** 3

*Fast and Ultrafast Spectroscopy* 3

*Computational Details* 4

**Figures S1-S12.** NMR spectra of compounds **1-3**. 5

**Figure S13-S15.** Spectrophotometric and spectrofluorimetric titrations of compounds **1** and **2**. 11

**Figure S16-S18.** Förster cycle of compounds **1-3**. 12

**Table S1.** Spectral properties of the differently protonated forms of compounds **1-3**. 14

**Figures S19-S20.** Optimized structures of zwitterionic/deprotonated compounds **1-3** in S_0_ and S_1_. 14

**Scheme S2.** Sketch of **2^+^** with labeled atoms. 15

**Tables S2-S3.** Bond lengths and bond angles for significant bonds in molecules **1-3.** 15

**Tables S4-S9.** Properties of compounds **1-3** calculated by the wB97XD/6-311+G(2d,p)//wB97XD/ 6-311+G(2d,p) model, together with the experimental absorption and emission maxima. 16

**Figures S21-S36.** Frontier molecular orbitals and effects of the S_0_→S_1_ and S_1_→ S_0_ transition on the electron density of compounds **1-3**. 17

**Figures S37-S38.** Femtosecond transient absorption measurements of compounds **2** and **3**. 27

**Table S10.** Rate constants for compounds **1-3** in buffered water. 28

**Figures S39-S41.** Femtosecond fluorescence up-conversion measurements of compounds **1-3**. 29

**Table S11.** Femtosecond transient absorption and fluorescence up-conversion results of compounds **1-3**. 31

**Figure S42.** TRANES analysis of femtosecond fluorescence up-conversion data of
compound **2**. 32

**Figures S43-S46.** Nanosecond laser flash photolysis measurements of compounds **1-3**. 33

***References*** 35

***Experimental Section***

*Materials and Methods*

*General*

Reagents and solvents were obtained from Sigma-Aldrich/Merck or Alfa Aesar/Thermo Scientific and used as received. Thin-layer chromatography was performed on Merck silica gel plates with the F-254 indicator. ^1^H and ^13^CNMR spectra were recorded at 27-40°C using a Varian Inova 500 spectrometer. Chemical shifts (*δ*) are expressed in ppm and referenced to the residual undeuterated solvent. MS spectra were recorded on an API 2000™ LC/MS/MS System.

*General Synthetic Procedure*

The selected aldehyde (1.1 mmol) and 1,2-dimethylpyridin-1-ium iodide^[52]^ (235 mg, 1 mmol) were mixed in 2 mL ethanol; 200 μL of piperidine were added, and the mixture was refluxed for 10-24h. The crude solid was isolated and washed with ethanol and diethyl ether; finally, the residue was recrystallized from ethanol to give the pure compound.

**Scheme S1.** Synthesis of compounds **1-3**.

*(E)-4-(2-(1-methylpyridin-1-ium-2-yl)vinyl)phenol iodide (****1****)*

Following the general procedure, the reaction of 4-hydroxybenzaldehyde gave product **2** as an orange solid (146 mg, 76%) ^1^H NMR *δ*H (D_2_O, 500 MHz, 27 °C), 8.59 (1H, d, *J* = 6.3 Hz, H_Py_), 8.38 (1H, t, *J* = 7.9 Hz; H_Py_); 8.26 (1H, d, *J* = 8.3 Hz, H_Py_), 7.76 (1H, t, *J* = 6.9 Hz; H_Py_), 7.70 (1H, d, J = 16.0 Hz, =CH-), 7.70 (2H, d, *J* = 8.7 Hz; H_Ph_); 7.26 (1H, d, *J* = 16.0 Hz, =CH), 6.98 (1H, t, *J* = 8.6 Hz; H_Ph_), 4.13 (3H, s, -CH_3_). ^13^C NMR *δ*C (D_2_O, 125 MHz, 27 °C), 153.52, 145.13, 144.29, 143.43, 130.57, 124.99, 124.60, 116.29, 114.14, 114.12, 114.10, 45.77. ESI-MS Found: [M+H]^+^ 212.3; C_14_H_14_NO requires [M+H]^+^ 212.1.

*(E)-2-(2-(1-methylpyridin-1-ium-2-yl)vinyl)phenol iodide (****2****)*

Following the general procedure, the reaction of salicylaldehyde gave product **1** as orange solid (154 mg, 73%) ^1^H NMR *δ*H (D_2_O, 500 MHz, 27 °C), 8.49 (1H, d, *J* = 6.3 Hz, H_Py_), 8.28 (1H, t, *J* = 7.9 Hz; H_Py_); 8.19 (1H, d, *J* = 8.0 Hz, H_Py_), 7.81 (1H, d, J = 16.1 Hz, =CH-), 7.65 (1H, t, *J* = 6.4 Hz; H_Py_), 7.62 (1H, dd, *J* = 7.9, 1.0 Hz; H_Ph_); 7.42 (1H, d, *J* = 16.1 Hz, =CH), 7.27 (1H, t, *J* = 7.1,1.4 Hz; H_Ph_), 6.94 (1H, t, *J* = 7.4 Hz; H_Ph_), 6.89 (1H, t, *J* = 7.1,1.0 Hz; H_Ph_), 4.13 (3H, s, -CH_3_). ^13^C NMR *δ*C (D_2_O, 125 MHz, 27 °C), 155.28, 153.53, 145.11, 144.35, 138.67, 132.35, 129.03, 125.11, 124.82, 121.96, 120.86, 117.18, 116.38, 45.71. ESI-MS Found: [M+H]^+^ 212.4; C_14_H_14_NO requires [M+H]^+^ 212.1.

*(E)-4-chloro-2-(2-(1-methylpyridin-1-ium-2-yl)vinyl)phenol iodide (****3****)*

Following the general procedure, the reaction of 4-chloro-2-hydroxybenzaldehyde gave product **3** as an orange/red solid (217 mg, 88%). ^1^H NMR *δ*H (D_2_O, 500 MHz, 40 °C), 8.50 (1H, d, *J* = 6.3 Hz, H_Py_), 8.27 (1H, t, *J* = 7.9 Hz; H_Py_); 8.13 (1H, d, *J* = 8.1 Hz, H_Py_), 7.67 (1H, d, J = 16.1 Hz, =CH-), 7.67 (1H, t, *J* = 6.4 Hz; H_Py_), 7.56 (1H, d, *J* = 2.6 Hz; H_Ph_); 7.34 (1H, d, *J* = 16.1 Hz, =CH), 7.15 (1H, dd, *J* = 8.8,2.6 Hz; H_Ph_), 6.78 (1H, d, *J* = 8.2 Hz; H_Ph_), 4.17 (3H, s, -CH_3_). ^13^C NMR *δ*C (D_2_O, 125 MHz, 27 °C), 154.11, 152.62, 145.21, 144.40, 137.18, 131.50, 128.00, 125.15, 124.82, 124.73, 123.01, 117.57, 117.51, 45.72. ESI-MS Found Found: [M+H]^+^ 246.6; C_14_H_13_NOCl requires [M+H]^+^ 246.1.

*Absorption and Emission Measurements*

Absorption spectra were measured with a Cary 4E (Varian) spectrophotometer, while fluorescence spectra, with appropriate corrections for the instrumental response, were detected by a FluoroMax-4P spectrofluorimeter (HORIBA Scientific) operated by FluorEssence. The fluorescence quantum yields (φ_F_, experimental error ± 10%) of dilute solutions (10^-6^ M) of the molecules were obtained by exciting each sample at the relative maximum absorption wavelength by employing tetracene or 9,10-diphenylanthracene (φ_F_ = 0.17 and 0.73, respectively, in air-equilibrated cyclohexane) as reference compounds.^[53]^

Spectrophotometric and fluorimetric titrations for the pKa and pKa* determination in water were performed by preparing a stock solution of each compound in deionized water and then diluting it in buffered solutions in a wide range of acidity, going from negative H_0_ values to pH = 12. Acidic conditions with a pH lower than 2 were obtained by resorting to perchloric acid (HClO_4_ 70%), while pH values ranging from 2 to 12 were obtained using Britton buffer solutions. Britton buffers were prepared by mixing an acid solution (H_3_BO_3_ 0.04 M, H_3_PO_4_ 0.04 M, and CH_3_COOH 0.04 M) with a 0.2 M solution of NaOH, adjusting the ionic strength of the final solution to 5 mM.

*Förster cycle for determining pKa**

An approximate estimation of the pKa* can also be carried out by resorting to the method proposed by Förster^[54,55]^ and Weller^[56-59]^ and known as the Förster cycle. The value of the pKa* can be determined by using the following equation: ΔpKa = pKa* − pKa = 0.625(Δν/T), where pKa is the known value for the acid/base equilibrium in the ground state, Δν is the energy difference (in cm^−1^) between the 0,0 transitions of the protonated/cationic and deprotonated/zwitterionic forms, measured from the intersection point of the normalized absorption and fluorescence spectra of the two species, and T is the absolute temperature in K.

*Fast and Ultrafast Spectroscopy*

Ultrafast time-resolved transient absorption and fluorescence up-conversion measurements were carried out by using Helios and Halcyone setups (Ultrafast System), already described elsewhere.^[60,61]^ Femtosecond excitation pulses at 400 nm of ca. 40 fs were generated by using an amplified Ti:sapphire laser system. In the transient absorption setup, the pump pulses were passed through a chopper that cut out every second pulse and collimated to the sample in a 2 mm quartz cuvette. Probe pulses for optical measurements were produced by passing a small portion of 800 nm light to an optical delay line with a time window of 3200 ps and focusing it into either a 2 mm thick sapphire crystal to generate a white-light continuum in the 470–800 nm range or a CaF_2_ crystal to obtain a white light enriched in the blue portion of the visible spectrum (420-660 nm). The white light was focused onto the sample, and the differential absorbance (ΔA) in the presence and absence of pump excitation was revealed by a CCD detector at each delay. In the up-conversion setup, the 400-nm pulse excites the sample, whereas the fundamental laser beam acts as the “gate” light, after passing through a delay line, which is then summed to the sample emission, promoting the up-conversion process in a motorized BBO crystal. The time resolution is about 200 fs, while the spectral resolution is 1.5 nm. All the measurements were carried out under magic-angle conditions. To avoid photoproduct interferences, the solutions were stirred during the experiments, and photodegradation was checked by recording the absorption spectrum of the samples before and after the measurements. Transient absorption and broadband fluorescence up-conversion data were analyzed using the Surface Xplorer PRO (Ultrafast Systems) software, which allows performing singular value decomposition of the 3D surface into principal components (spectra and kinetics) and global analysis (giving lifetimes and Decay Associated Spectra (DAS) of the detected transients). Evolution Associated Spectra (EAS) were calculated by performing global analysis according to a consecutive model for the detected transients by means of the GloTarAn software.^[62]^

Laser flash photolysis (Edinburgh LP980) experiments were performed upon excitation at 355 nm (third harmonic of a Quanta-Ray/ INDI Nd:YAG laser, Spectra Physics) with nanosecond time-resolution (pulse width 7 ns and laser energy < 1 mJ per pulse) coupled with a PMT for signal detection. The excitation at 355 nm was the pump pulse, while a pulsed xenon lamp was used to probe the absorption properties of the produced transients in the 375-600 nm range. Measurements were performed either in air-equilibrated solutions or by purging the sample with pure molecular nitrogen to take into account the effect of molecular oxygen. Experiments in the presence of a proton scavenger were conducted in a 0.01 M acetate solution in buffered water at pH 6.

*Computational Details*

Quantum-mechanical calculations were carried out using the Gaussian 09 package.^[63]^ Density functional theory (DFT) based on the wB97XD method was used to optimize the geometry and to obtain the properties of the compounds in the ground state, while the lowest excited singlet states were characterized by time-dependent (TD) DFT wB97XD excited-state calculations.^[64,65]^ In both cases, a 6-311+G(2d,p) basis set was employed. Water solvation effects were included in the calculations by means of the conductor-like polarizable continuum model (CPCM).^[66]^


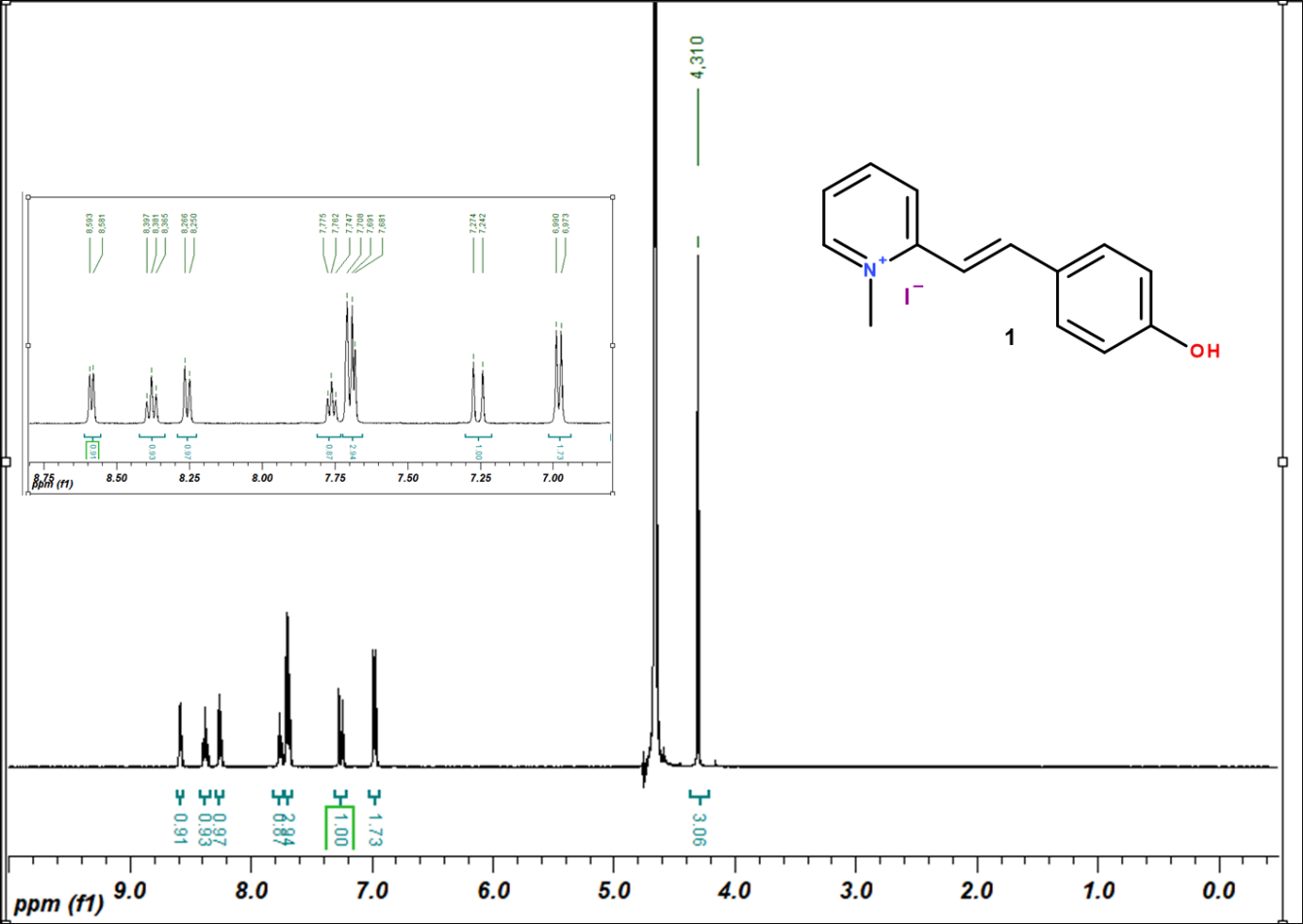


**Figure S1.** ^1^H-NMR spectrum of **1** (D_2_O, 500 MHz, 27 °C)


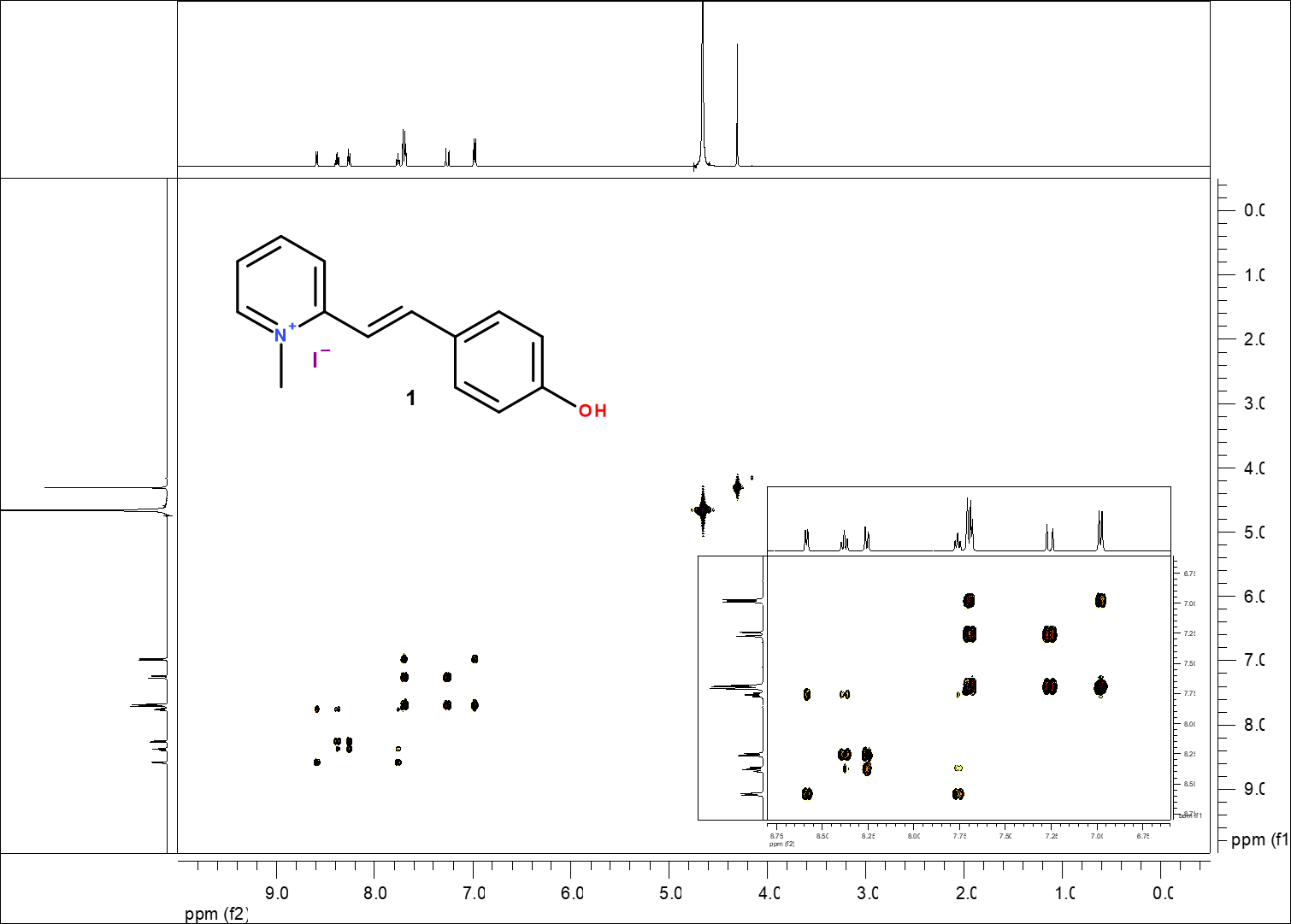


**Figure S2.** ^1^H-^1^H gCOSY spectrum of **1** (D_2_O, 500 MHz, 27 °C)


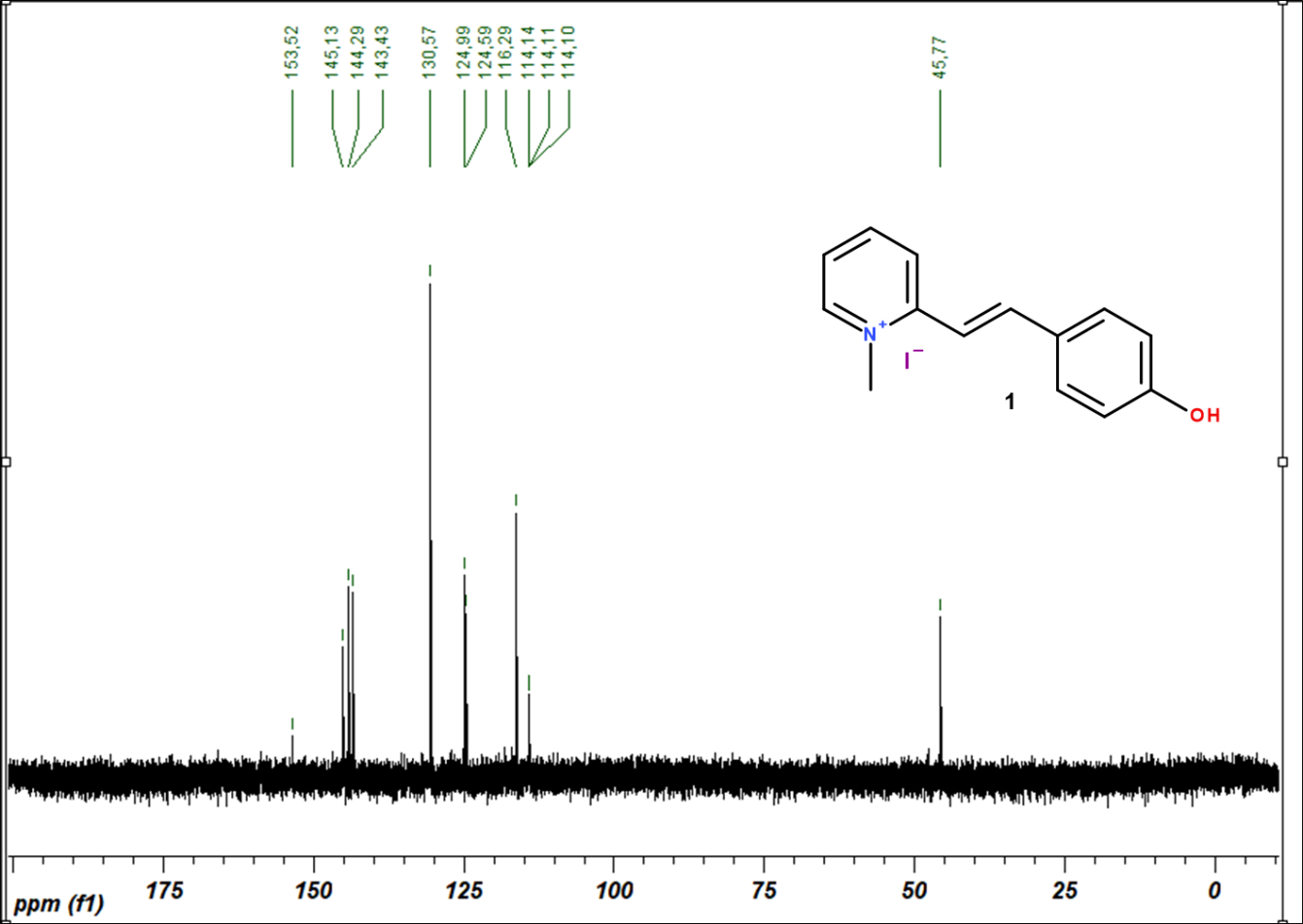


**Figure S3.** ^13^C-NMR spectrum of **1** (D_2_O, 500 MHz, 27 °C)


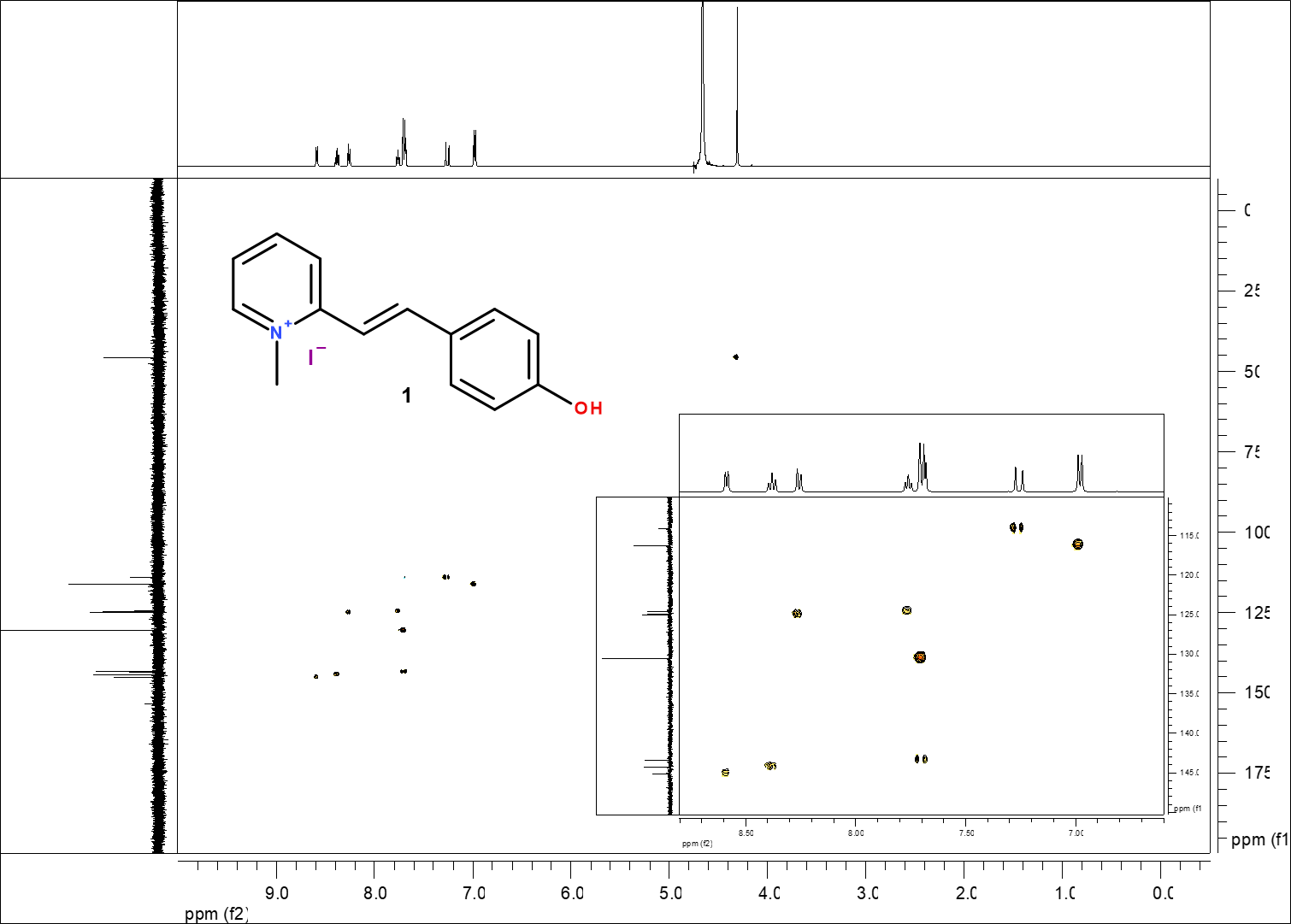


**Figure S4.** ^1^H-^13^C gHSQCAD spectrum of **1** (D_2_O, 500 MHz, 27 °C)

**
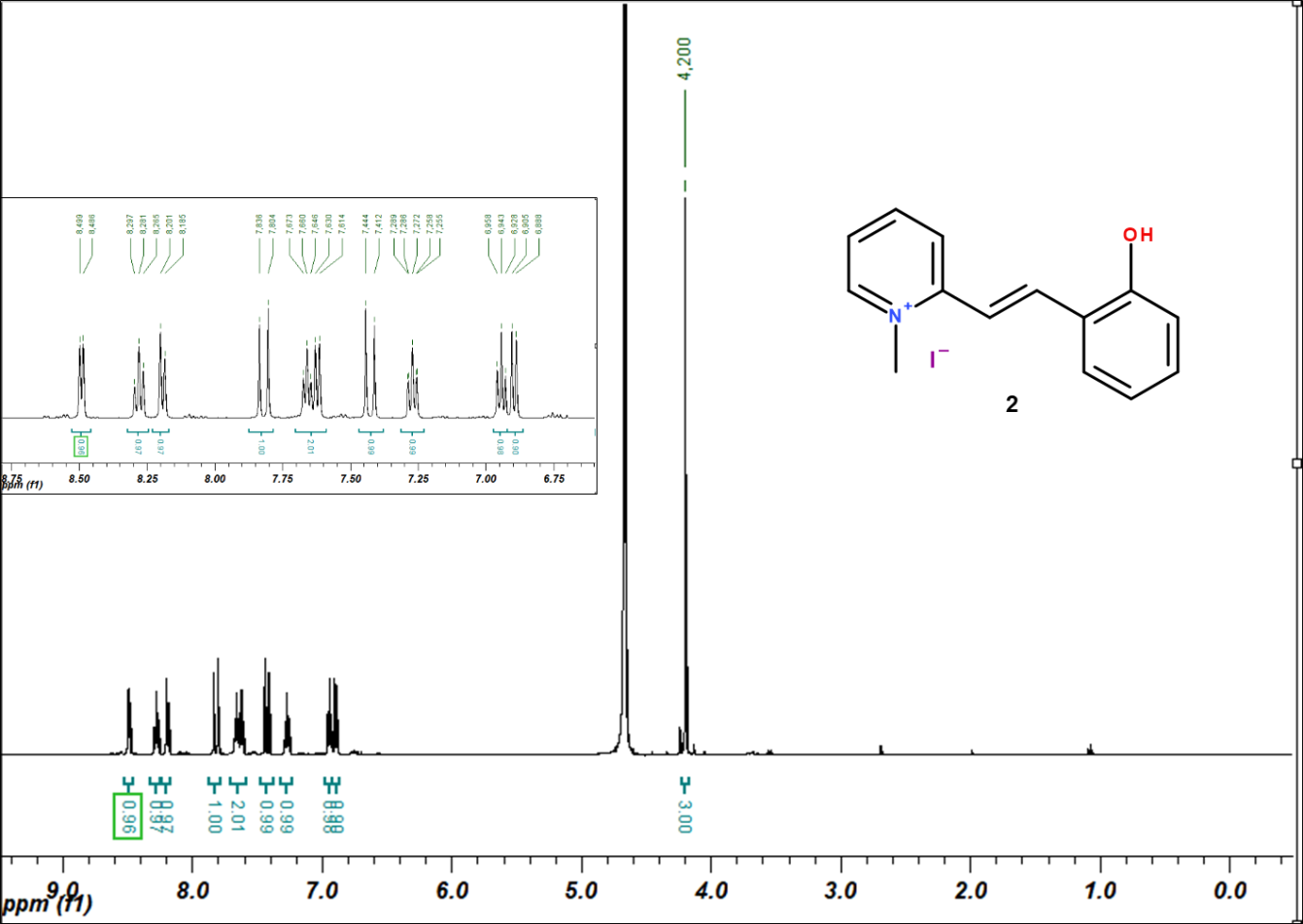
**

**Figure S5.** ^1^H-NMR spectrum of **2** (D_2_O, 500 MHz, 27 °C)


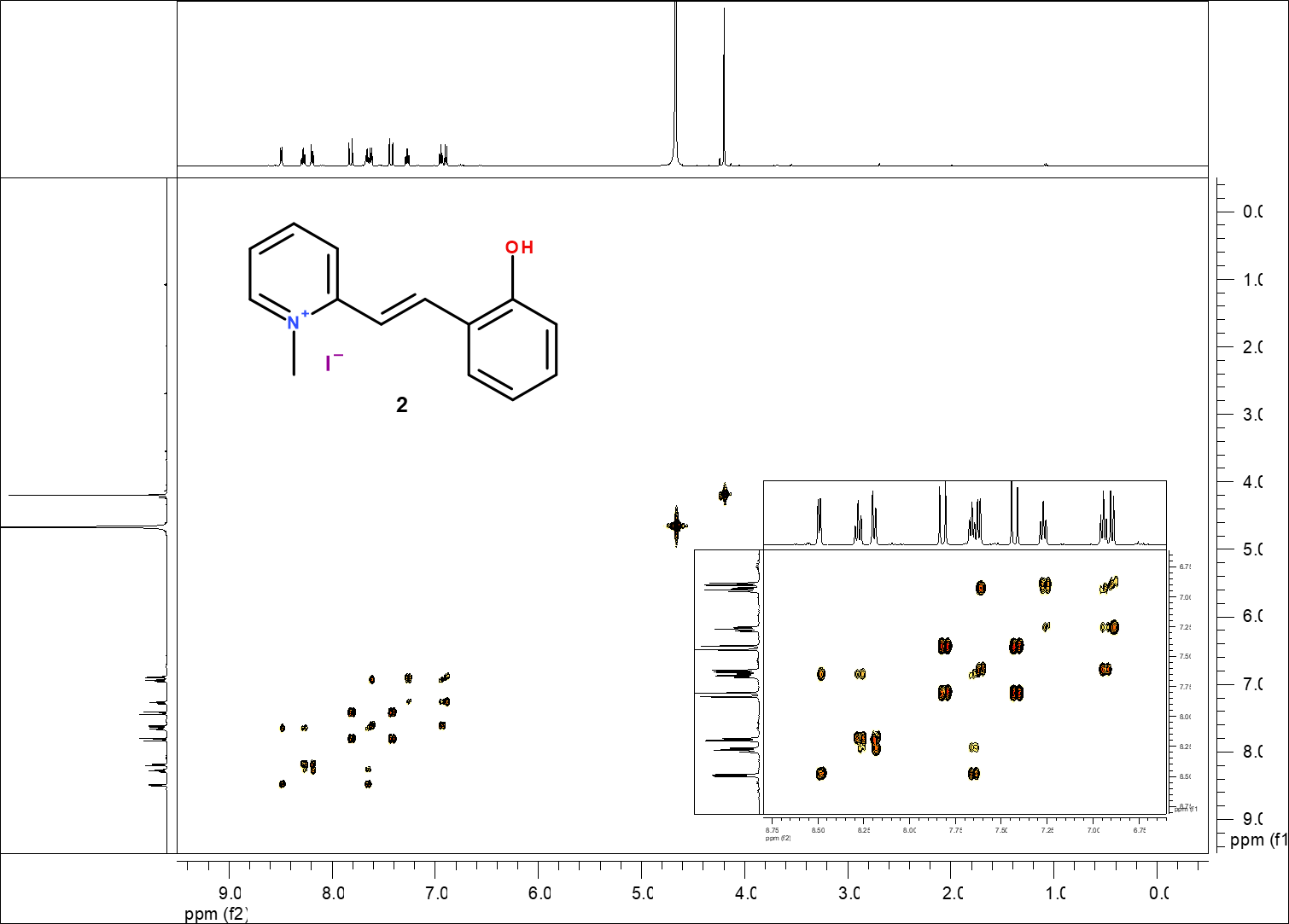


**Figure S6.** ^1^H-^1^H gCOSY spectrum of **2** (D_2_O, 500 MHz, 27 °C)


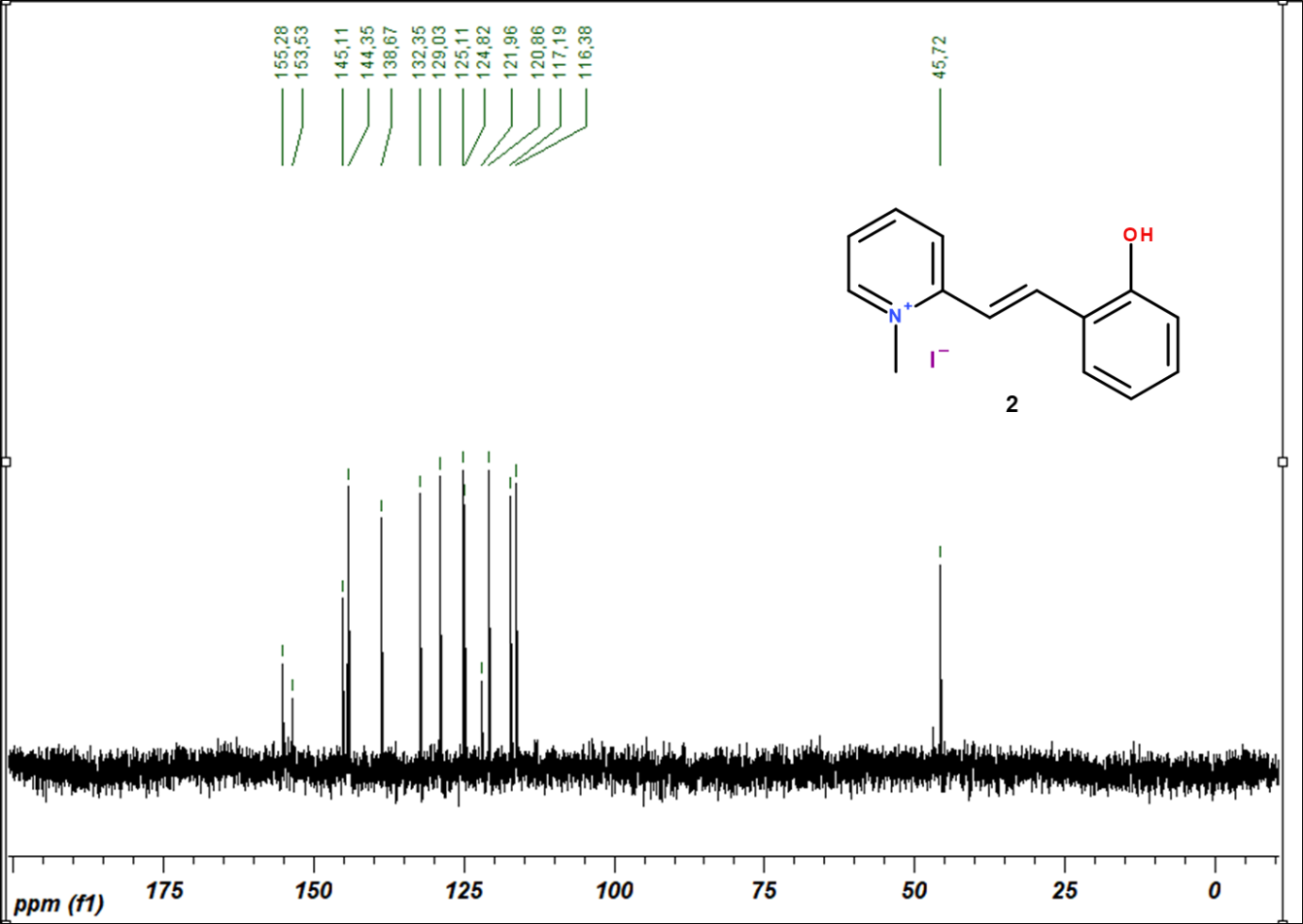


**Figure S7.** ^13^C-NMR spectrum of **2** (D_2_O, 500 MHz, 27 °C)


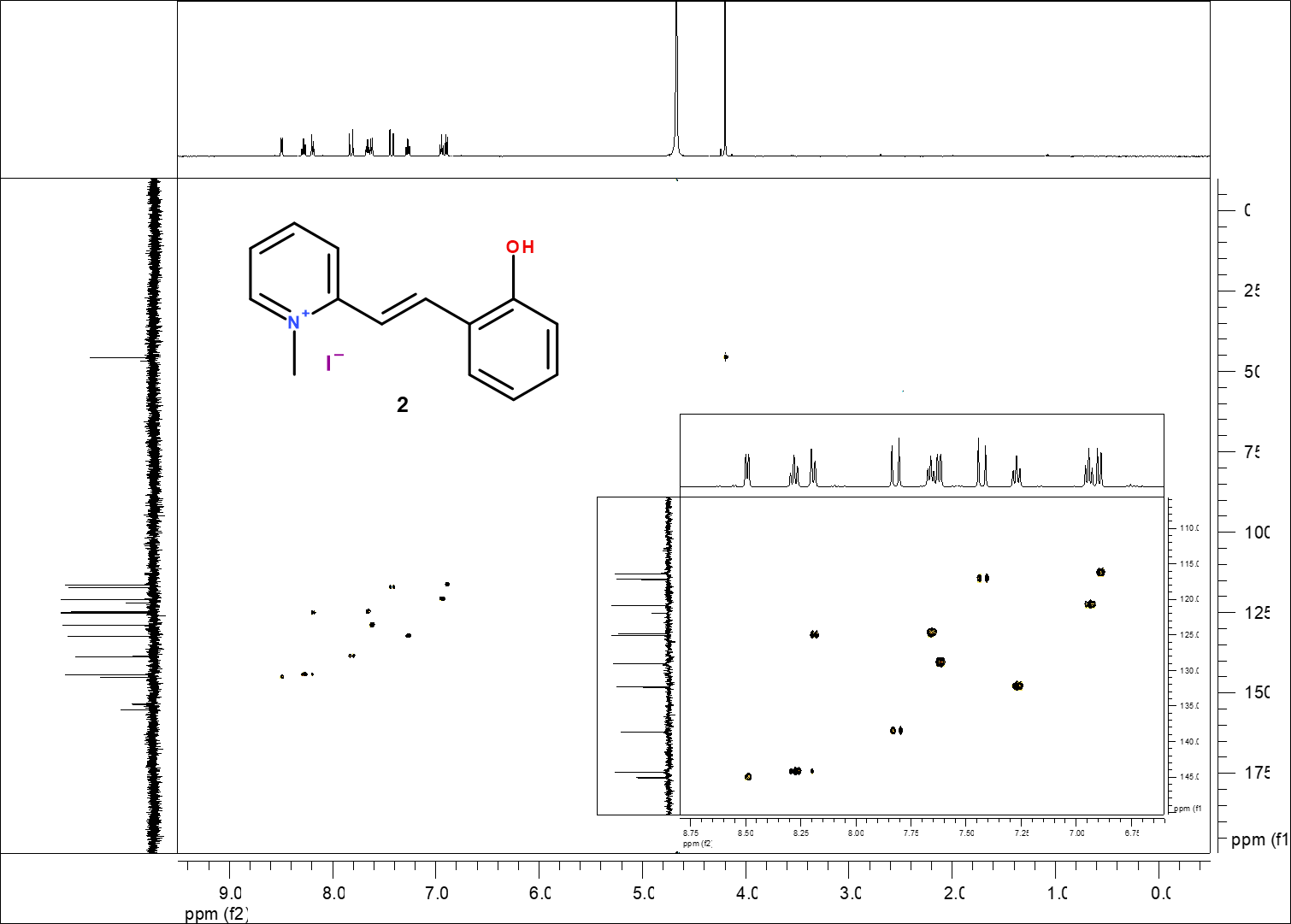


**Figure S8.** ^1^H-^13^C gHSQCAD spectrum of **2** (D_2_O, 500 MHz, 27 °C)


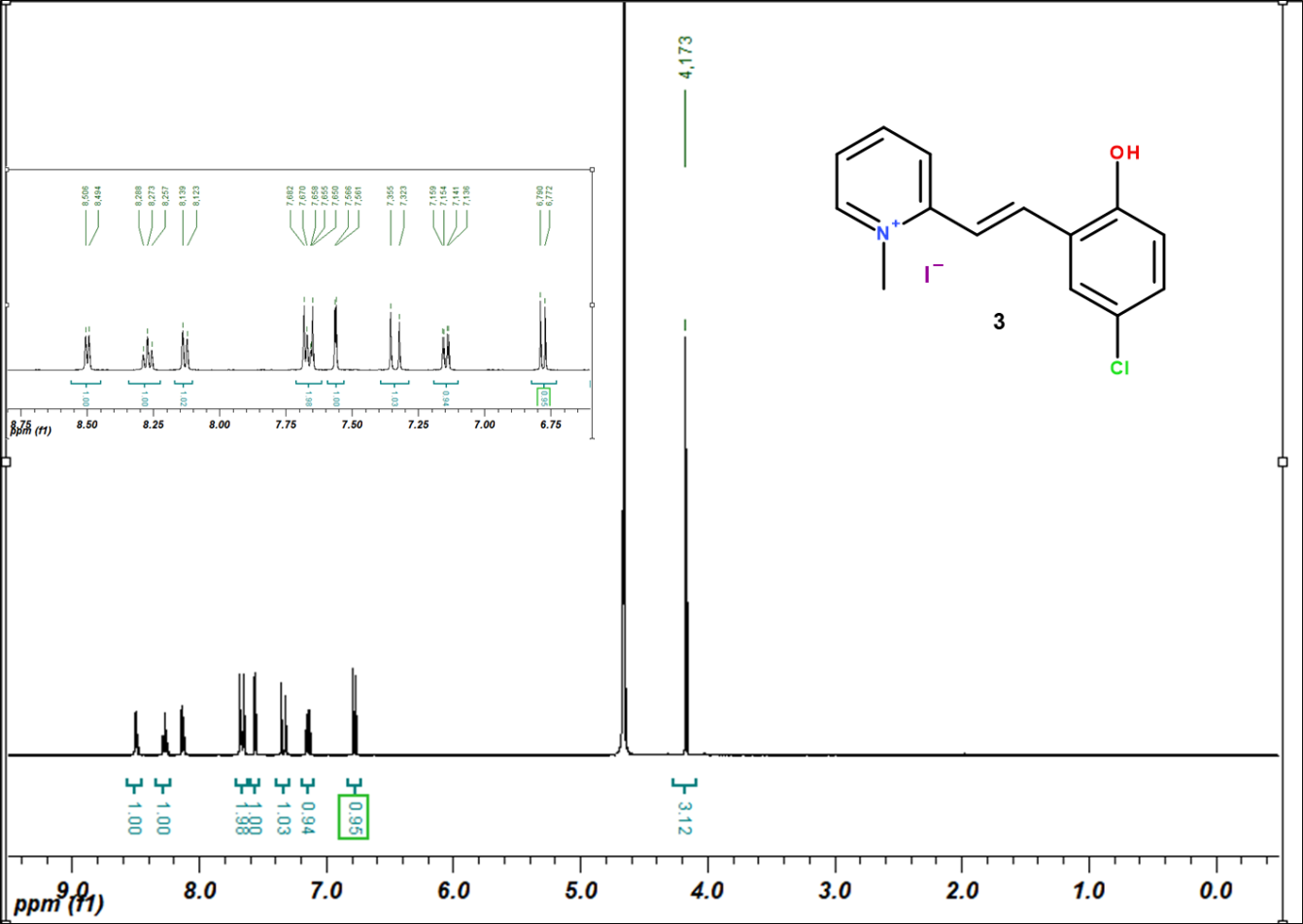


**Figure S9.** ^1^H-NMR spectrum of **3** (D_2_O, 500 MHz, 40 °C)


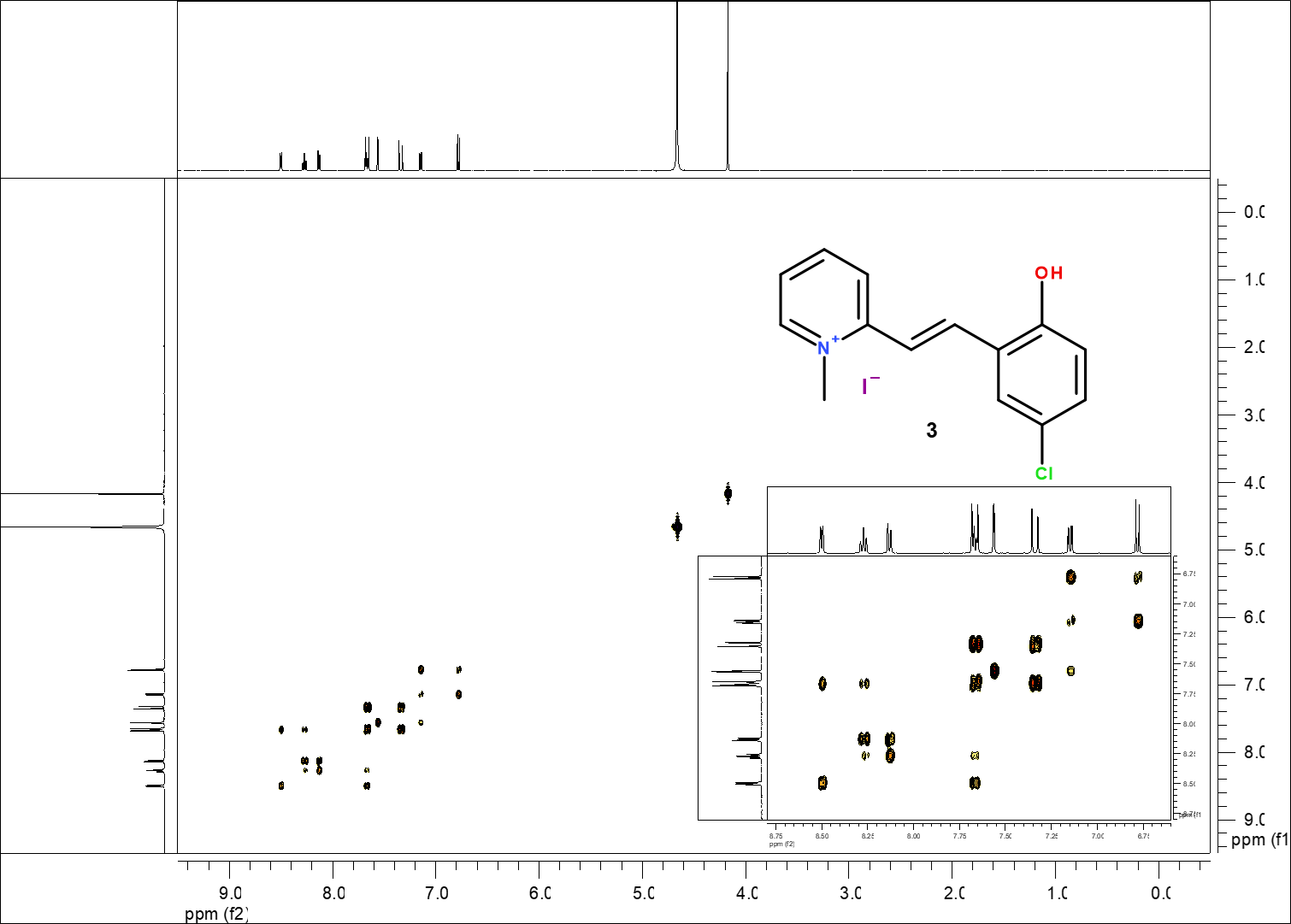


**Figure S10.** ^1^H-^1^H gCOSY spectrum of **3** (D_2_O, 500 MHz, 40 °C)


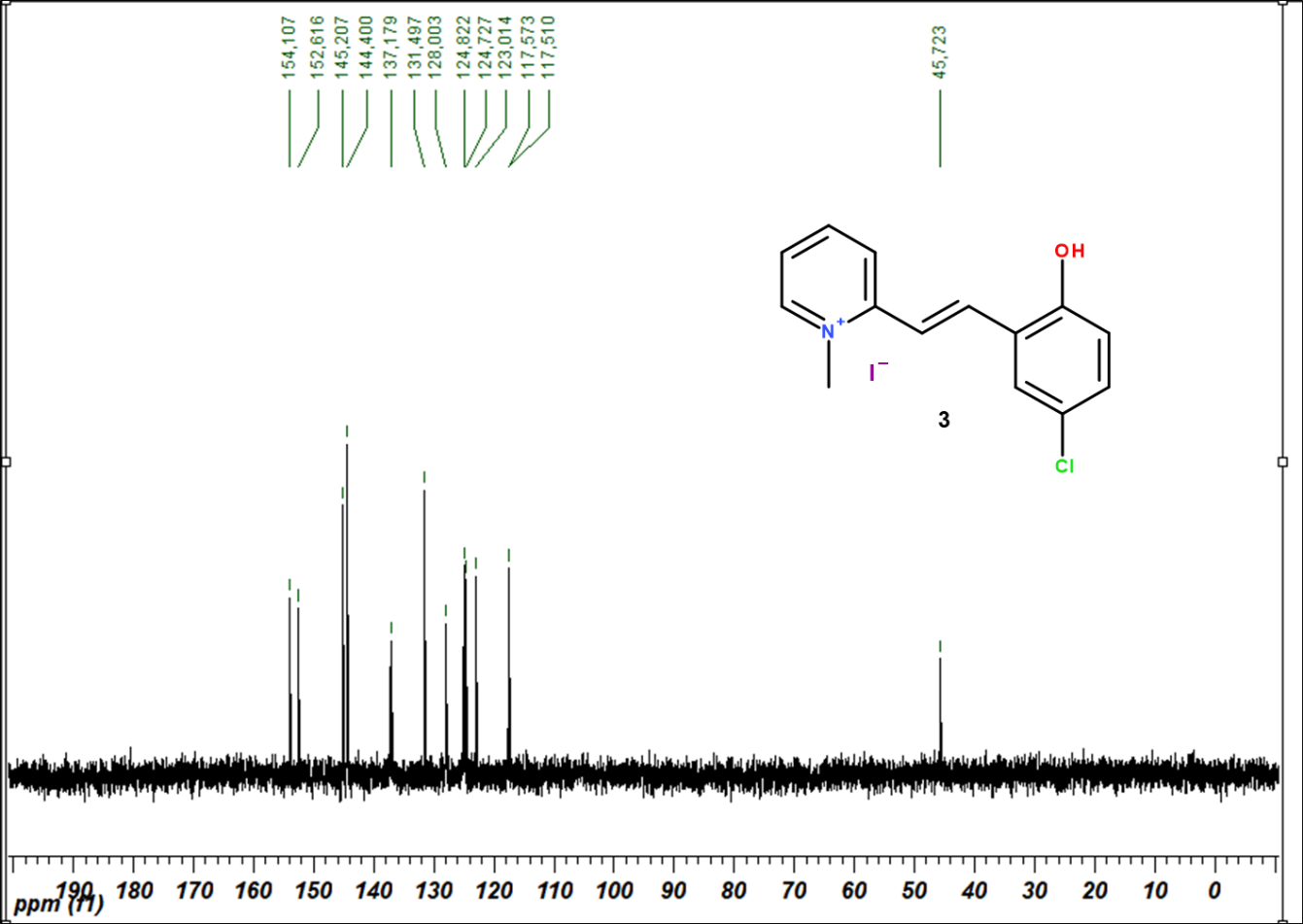


**Figure S11.** ^13^C-NMR spectrum of **3** (D_2_O, 500 MHz, 40 °C)


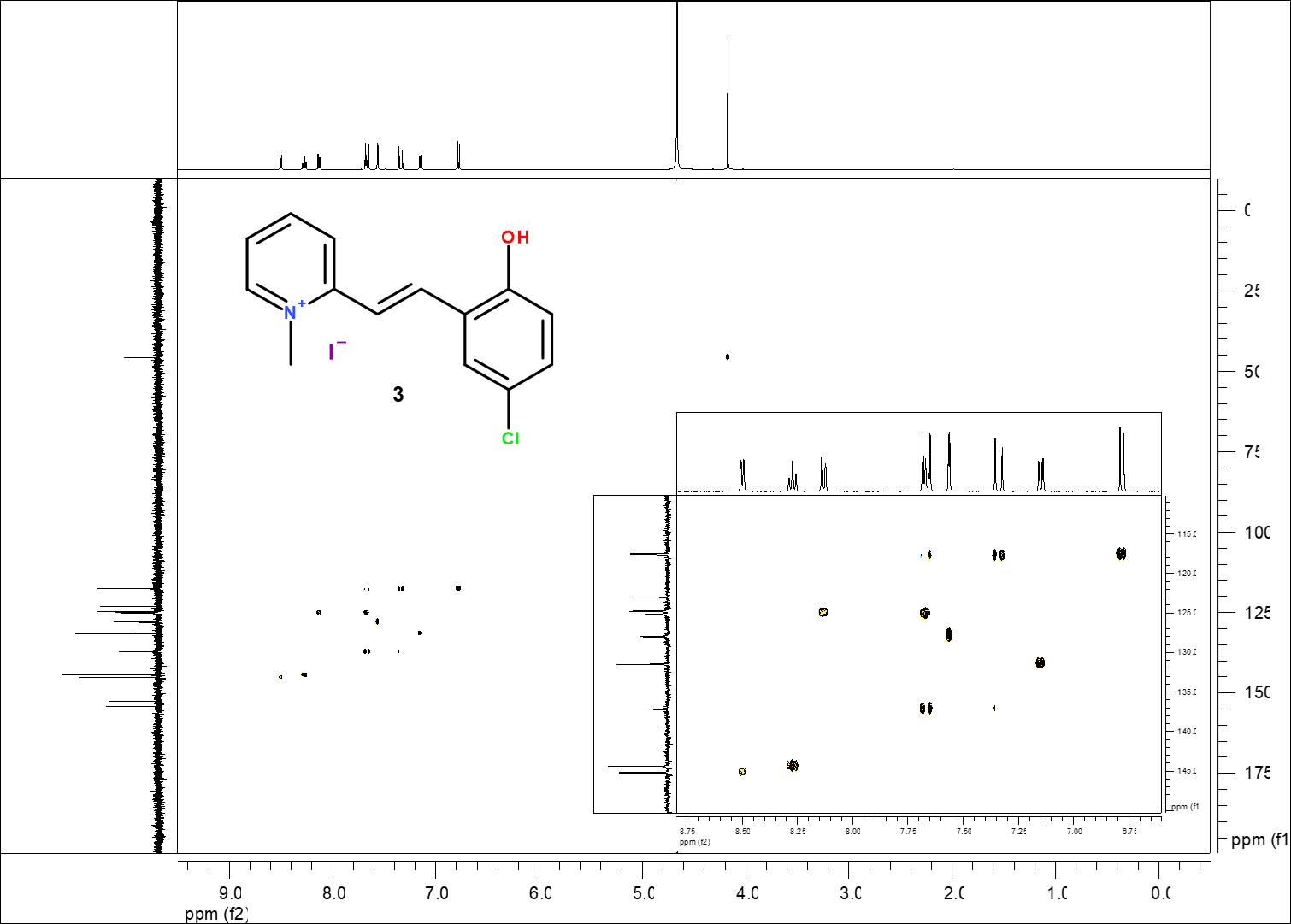


**Figure S12.** ^1^H-^13^C gHSQCAD spectrum of **3** (D_2_O, 500 MHz, 40 °C)

**Figure S13.** Spectrophotometric titration of compound **1** (left panel) and compound **2** (right panel) in a large pH interval. Arrows point to changes in the absorption spectrum as the pH decreases.

**Figure S14.** pKa fitting of the spectrophotometric titration of compounds **1** (left panel) and **2** (right panel) carried out at significant wavelengths correpsonding to the maxima of the protonated and deprotonated forms, green and red curves, respectively.

**Figure S15.** Spectrofluorimetric titrations of compound **2** (left panel) in a large pH/H_0_ interval. Inset: zoom-in at low emission intensities to appreciate the changes in the emission between pH 12 and 5. Arrows point to changes in the emission spectrum as the pH/H_0_ decreases. Relative pKa/pKa* fitting (right panel) obtained by plotting the areas subtended by the emission spectra as a function of [H^+^].

**Förster cycle of 1**

H_0_ −3 (ACID) pH 12 (BASE)

ν_0−0_ (ACID) = 425 nm

ν_0−0_ (BASE) = 490 nm

ΔE = ν_0−0_ (ACID) − ν_0−0_ (BASE) = 3120 cm^−1^  pKa* = 0.625 * (−3120) / 298 + pKa = **2.0**

**Figure S16.** Estimation of the pKa*of **1** through the Förster cycle: panel A, normalized absorption and emission spectra of the protonated/cationic form of **1** recorded at H_0_ −3; panel B, normalized absorption and emission spectra of the deprotonated/zwitterionic form of **1** recorded at pH 12.

**Förster cycle of 2**

H_0_ −3 (ACID) pH 12 (BASE)

ν_0−0_ (ACID) = 415 nm

ν_0−0_ (BASE) = 515 nm

ΔE = ν_0−0_ (ACID) − ν_0−0_ (BASE) = 4680 cm^−1^  pKa* = 0.625 * (−4680) / 298 + pKa = **− 1.4**

**Figure S17.** Estimation of the pKa*of **2** through the Förster cycle: panel A, normalized absorption and emission spectra of the protonated/cationic form of **2** recorded at H_0_ −3; panel B, normalized absorption and emission spectra of the deprotonated/zwitterionic form of **2** recorded at pH 12.

**Förster cycle of 3**

pH −3 (ACID) pH 12 (BASE)

ν_0−0_ (ACID) = 428 nm

ν_0−0_ (BASE) = 534 nm

ΔE = ν_0−0_ (ACID) − ν_0−0_ (BASE) = 4640 cm^−1^  pKa* = 0.625 * (−4640) / 298 + pKa = **− 1.9**

**Figure S18.** Estimation of the pKa*of **3** through the Förster cycle: panel A, normalized absorption and emission spectra of the protonated/cationic form of **3** recorded at H_0_ −3; panel B, normalized absorption and emission spectra of the deprotonated/zwitterionic form of **3** recorded at pH 12.

**Table S1.** Spectral properties of the protonated/cationic and deprotonated/zwitterionic forms of compounds **1-3**.

| **Compound** | **λ_abs_ /nm** | **ε /M^−1^cm^−1^** | **λ_em_ /nm** | **ν_0−0_**  **/nm** |
| --- | --- | --- | --- | --- |
| **1^+^** | 362 | 26,900 | 485 | 425 |
| **1** | 430 | 31,500 | 560 | 490 |
| **2^+^** | 356 | 19,100 | 505 | 415 |
| **2** | 327, 438 | 15,900 | 640 | 515 |
| **3^+^** | 328, 360 | 20,500 | 524 | 428 |
| **3** | 322, 444 | 19,100 | 649 | 534 |

Absorption (λ_abs_) and emission (λ_em_) maxima (when two wavelengths are reported, the main maximum is underlined), molar extinction coefficient (ε) at the main absorption maximum, and energy of the 0,0 transition (ν_0−0_ ), measured as the intersection point of the normalized absorption and fluorescence spectra.

**1 2 3**

**Figure S19.** Optimized structures of zwitterionic/deprotonated compounds **1-3** obtained by
wB97XD/6-311+G(2d,p) @ S_0_ in water.


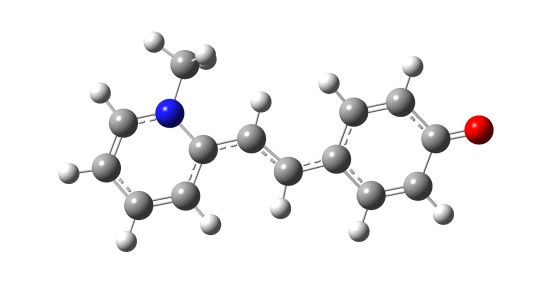

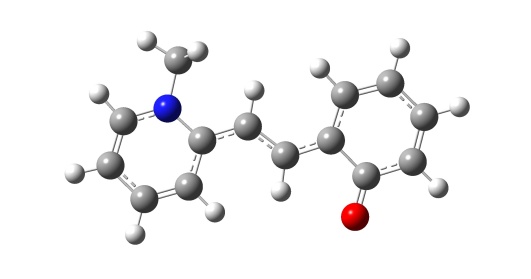

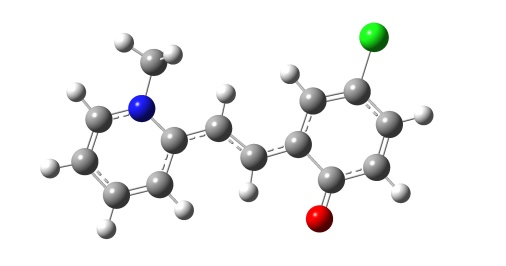


**1 2 3**

**Figure S20.** Optimized structures of zwitterionic/deprotonated compounds **1-3** obtained by
wB97XD/6-311+G(2d,p) @ S_1_ in water.

**
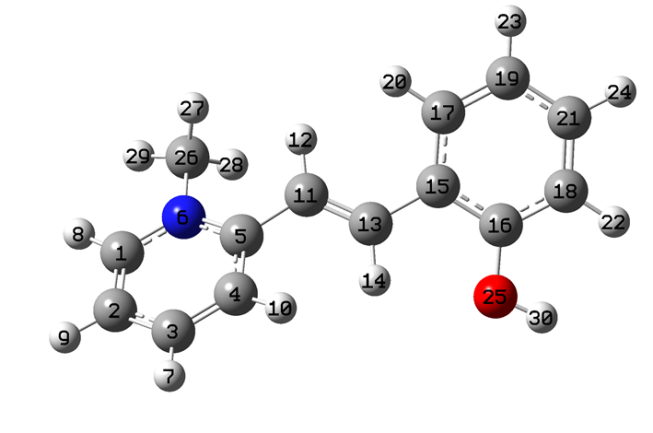
**

**Scheme S2.** Sketch of **2^+^** with labeled atoms, numbered from 1 to 30, as a representative example.

**Table S2.** Bond lengths and bond angles for significant bonds in molecules **1-3** obtained by wB97XD/6-311+G(2d,p) @ S_0_ in water.

| **S_0_** | Bond length (Å) | | | | | Bond angle (deg) | | | |
| --- | --- | --- | --- | --- | --- | --- | --- | --- | --- |
|  | 14-13 | 13-15 | 15-16 | 16-25^[a]^ | 14-25^[a]^ | 14-13-15 | 13-15-16 | 15-16-25^[a]^ | 13-14-25^[a]^ |
| **1** | 1.08678 | 1.41678 | 1.41733 | 1.08682 | 2.28134 | 113.96 | 119.17 | 118.26 | 96.61 |
| **1^+^** | 1.08681 | 1.45703 | 1.39688 | 1.08440 | **2.32606** | 114.72 | 118.77 | 119.42 | 94.99 |
| **2** | 1.08601 | 1.42157 | 1.45383 | 1.26165 | 2.33602 | 112.71 | 117.20 | 123.05 | 103.69 |
| **2^+^** | 1.08465 | 1.45921 | 1.40356 | 1.35498 | **2.35063** | 115.28 | 119.10 | 117.68 | 99.33 |
| **3** | 1.08593 | 1.42564 | 1.45179 | 1.25997 | 2.33627 | 112.52 | 117.23 | 123.14 | 103.78 |
| **3^+^** | 1.08462 | 1.46088 | 1.40272 | 1.35255 | **2.37105** | 115.32 | 119.28 | 117.62 | 98.26 |

[a] in the case of compound **1**, atom 25 is a hydrogen instead of an oxygen.

**Table S3.** Bond lengths and bond angles for significant bonds in molecules **1-3** obtained by wB97XD/6-311+G(2d,p) @ S_1_ in water.

| **S_1_** | Bond length (Å) | | | | | Bond angle (deg) | | | |
| --- | --- | --- | --- | --- | --- | --- | --- | --- | --- |
|  | 14-13 | 13-15 | 15-16 | 16-25^[a]^ | 14-25^[a]^ | 14-13-15^[a]^ | 13-15-16 | 15-16-25^[a]^ | 13-14-25^[a]^ |
| **1** | 1.08369 | 1.41483 | 1.43263 | 1.08569 | 2.32844 | 115.93 | 119.24 | 118.01 | 94.74 |
| **1^+^** | 1.08295 | 1.39692 | 1.43476 | 1.08367 | **2.32743** | 116.29 | 119.12 | 118.54 | 94.68 |
| **2** | 1.08150 | 1.41543 | 1.48452 | 1.25248 | 2.31537 | 114.89 | 116.70 | 119.99 | 102.05 |
| **2^+^** | 1.08027 | 1.39600 | 1.45120 | 1.32904 | **2.28813** | 116.74 | 118.84 | 115.94 | 100.73 |
| **3** | 1.08173 | 1.41286 | 1.48521 | 1.24942 | 2.31587 | 114.73 | 116.83 | 120.04 | 102.12 |
| **3^+^** | 108036 | 1.39506 | 1.45155 | 1.32441 | **2.29265** | 116.69 | 118.99 | 115.98 | 100.58 |

[a] in the case of compound **1**, atom 25 is a hydrogen instead of an oxygen.

**Table S4.** Absorption wavelengths (λ), oscillator strength (f), and molecular orbitals of **1** in water (CPCM) calculated by the wB97XD/6-311+G(2d,p)//wB97XD/6-311+G(2d,p) model, together with the experimental absorption and emission maxima.

| Transition | λ_th_/nm | f | MO | % | λ_exp_/nm |
| --- | --- | --- | --- | --- | --- |
| S_0_→T_1_ | 771 | 0.0000 | π_H_→π_L_* | 94 |  |
| S_0_→S_1_ | 498 | 1.6350 | π_H_→π_L_* | 93 | 430 |
| S_0_→T_2_ | 414 | 0.0000 | π_H_→π_L+1_* | 60 |  |
| S_0_→T_3_ | 363 | 0.0000 | π_H-2_→π_L_* | 50 |  |
| S_0_→T_4_ | 344 | 0.0000 | π_H_→π_L+2_*  π_H_→π_L+5_* | 36  31 |  |
| S_0_→S_2_ | 325 | 0.0386 | π_H_→π_L+1_* | 85 |  |
| S_0_→T_5_ | 313 | 0.0000 | n_H-1_→π_L_* | 69 |  |
| S_0_→T_6_ | 296 | 0.0000 | π_H-3_→π_L_*  π_H-3_→π_L+2_*  π_H_→π_L+2_* | 29  20  21 |  |
| S_0_→S_3_ | 294 | 0.0000 | n_H-1_→π_L_* | 73 |  |
| S_0_→T_7_ | 283 | 0.0000 | π_H-3_→π_L_*  π_H_→π_L+5_* | 34  31 |  |
| S_0_→S_4_ | 282 | 0.0674 | π_H_→π_L+2_* | 67 |  |
| S_0_→T_8_ | 268 | 0.0000 | π_H-5_→π_L_*  π_H-4_→π_L_*  π_H-4_→π_L+1_* | 21  19  33 |  |
| S_0_→T_9_ | 263 | 0.0000 | π_H-3_→π_L+2_*  π_H-3_→π_L+5_* | 32  19 |  |
| S_0_→S_5_ | 256 | 0.0164 | π_H-2_→π_L_* | 50 |  |
| S_0_→T_10_ | 253 | 0.0000 | π_H-4_→π_L_*  π_H_→π_L+13_* | 20  14 |  |
| S_0_→S_6_ | 252 | 0.0653 | π_H-3_→π_L_* | 63 |  |
| S_0_→S_7_ | 242 | 0.0157 | π_H_→π_L+3_* | 43 |  |
| S_0_→S_8_ | 234 | 0.0602 | π_H_→π_L+5_* | 53 |  |
| S_0_→S_9_ | 221 | 0.0002 | π_H_→π_L+7_* | 42 |  |
| S_0_→S_10_ | 221 | 0.0055 | π_H_→π_L+4_* | 44 |  |
| S_1_→S_0_ | 535 | 1.6269 | π_H_→π_L_* | 95 | 560 |


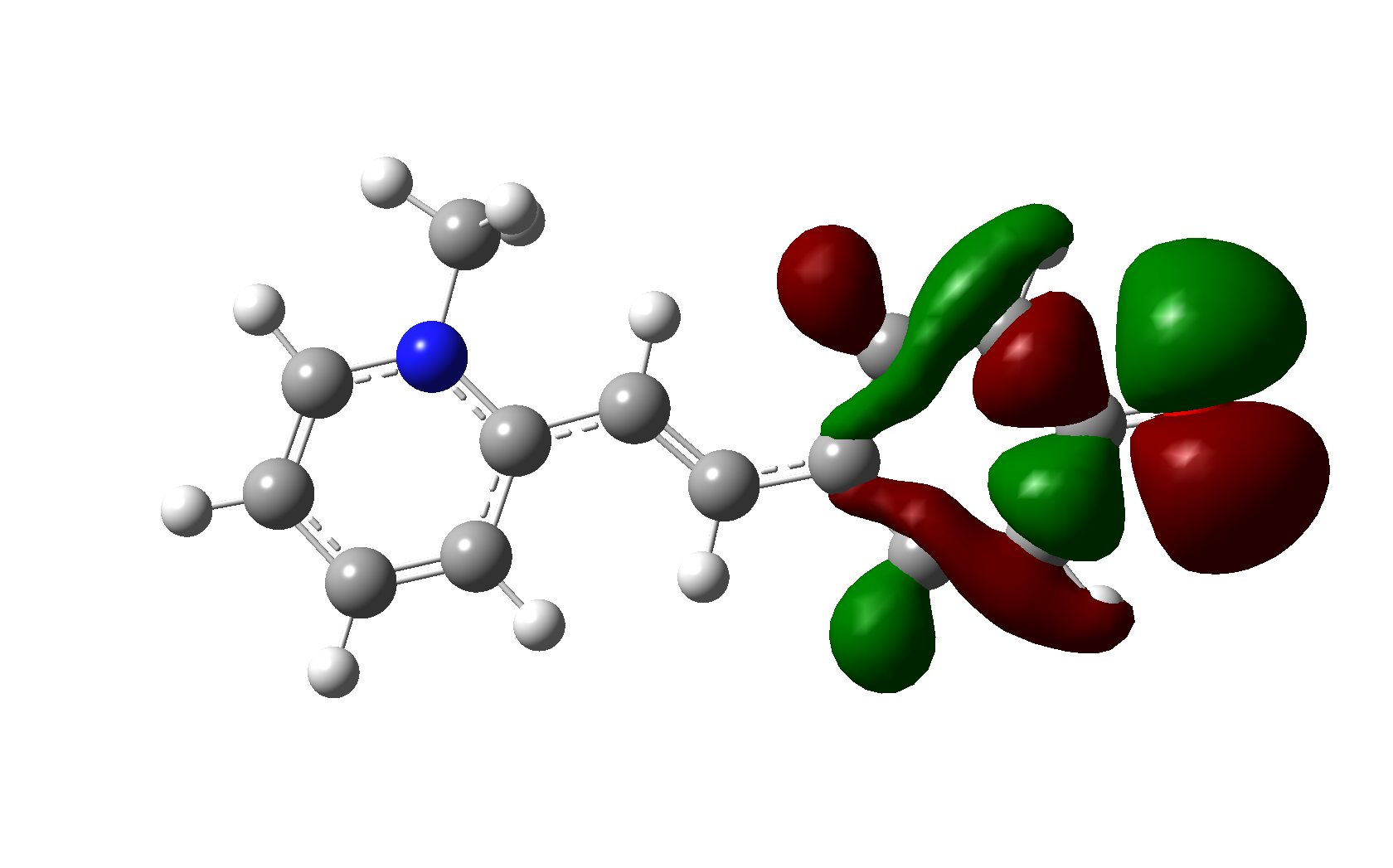


π_H−1_ π_H_

__

π_L_ π_L+1_

**Figure S21.** Frontier molecular orbitals of **1**.

**Figure S22.** Effect of the S_0_→S_1_ transition on the electron density of **1**; increase and decrease of electron densities are represented by blue (+0.0001) and red (−0.0001), respectively.

**
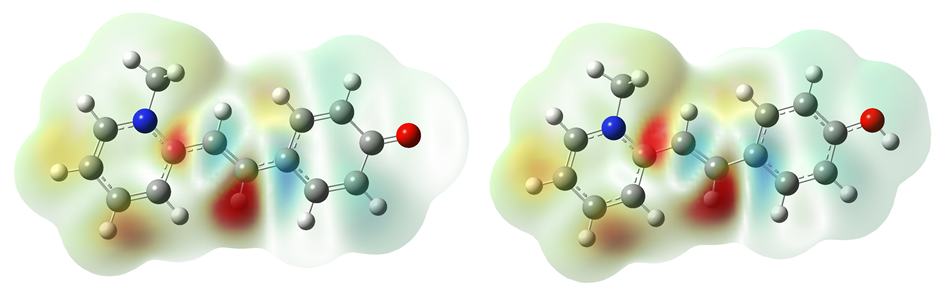
**

**Figure S23.** Effect of the S_1_→S_0_ transition on the electron density of **1**; increase and decrease of electron densities are represented by blue (+0.0001) and red (−0.0001), respectively.

**Table S5.** Absorption wavelengths (λ), oscillator strength (f), and molecular orbitals of **1^+^** in water (CPCM) calculated by the wB97XD/6-311+G(2d,p)//wB97XD/6-311+G(2d,p) model, together with the experimental absorption and emission maxima.

| Transition | λ_th_/nm | f | MO | % | λ_exp_/nm |
| --- | --- | --- | --- | --- | --- |
| S_0_→T_1_ | 524 | 0.0000 | π_H_→π_L_* | 71 |  |
| S_0_→S_1_ | 356 | 1.2769 | π_H_→π_L_* | 89 | 362 |
| S_0_→T_2_ | 342 | 0.0000 | π_H-1_→π_L_*  π_H_→π_L+1_* | 31  31 |  |
| S_0_→T_3_ | 328 | 0.0000 | π_H-4_→π_L_* | 27 |  |
| S_0_→T_4_ | 292 | 0.0000 | π_H-5_→π_L_* | 58 |  |
| S_0_→T_5_ | 284 | 0.0000 | π_H-2_→π_L_* | 44 |  |
| S_0_→T_6_ | 275 | 0.0000 | π_H-6_→π_L_*  π_H-5_→π_L+1_* | 25  20 |  |
| S_0_→T_7_ | 269 | 0.0000 | π_H-2_→π_L_*  π_H-2_→π_L+1_* | 24  28 |  |
| S_0_→S_2_ | 265 | 0.0495 | π_H-1_→π_L_* | 60 |  |
| S_0_→S_3_ | 257 | 0.0109 | π_H-2_→π_L_*  π_H-2_→π_L+1_* | 29  28 |  |
| S_0_→T_8_ | 256 | 0.0000 | π_H-3_→π_L_*  π_H_→π_L+3_* | 22  19 |  |
| S_0_→T_9_ | 240 | 0.0000 | π_H-2_→π_L+2_*  π_H_→π_L+2_* | 17  23 |  |
| S_0_→S_4_ | 231 | 0.0797 | π_H-3_→π_L_* | 38 |  |
| S_0_→S_5_ | 227 | 0.1978 | π_H-1_→π_L_* | 33 |  |
| S_0_→T_10_ | 215 | 0.0000 | π_H-4_→π_L+3_* | 59 |  |
| S_0_→S_6_ | 213 | 0.0589 | π_H-1_→π_L+1_* | 47 |  |
| S_0_→S_7_ | 200 | 0.1616 | π_H-2_→π_L_* | 53 |  |
| S_0_→S_8_ | 197 | 0.0411 | π_H-4_→π_L_*  π_H_→π_L+2_* | 41  32 |  |
| S_0_→S_9_ | 195 | 0.2420 | π_H-3_→π_L_* | 22 |  |
| S_0_→S_10_ | 191 | 0.2698 | π_H_→π_L+5_* | 35  44 |  |
| S_1_→S_0_ | 468 | 1.4080 | π_H_→π_L_* | 97 | 485 |


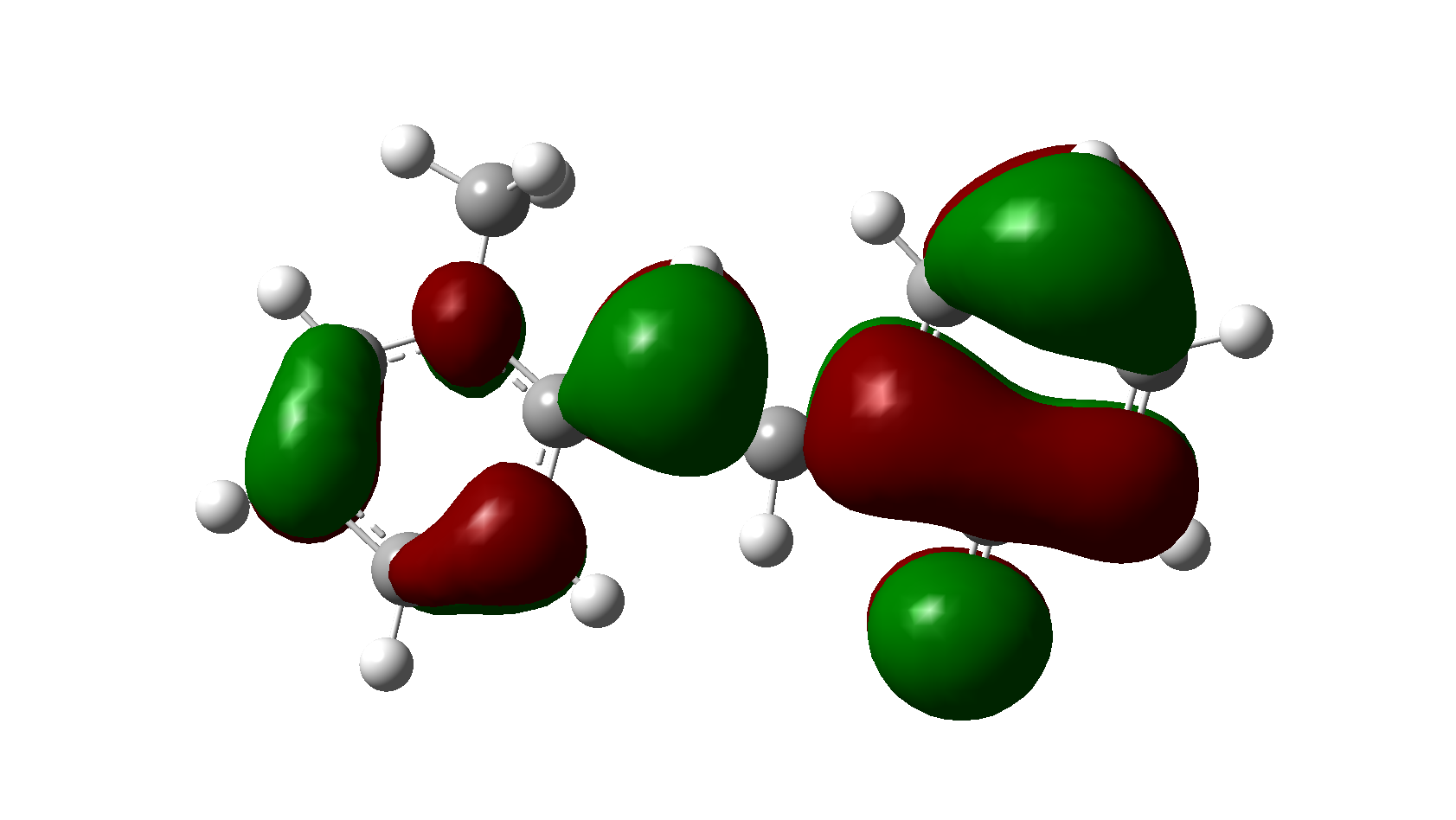


π_H−1_ π_H_

__

π_L_ π_L+1_

**Figure S24.** Frontier molecular orbitals of **1^+^**.

**Figure S25.** Effect of the S_0_→S_1_ transition on the electron density of **1^+^**; increase and decrease of electron densities are represented by blue (+0.0001) and red (−0.0001), respectively.


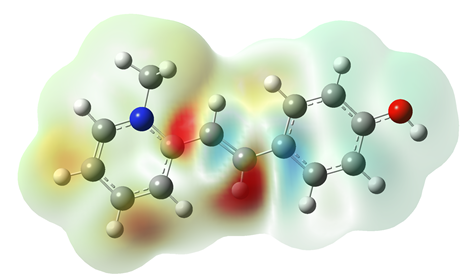


**Figure S26.** Effect of the S_1_→S_0_ transition on the electron density of **1^+^**; increase and decrease of electron densities are represented by blue (+0.0001) and red (−0.0001), respectively.

**Table S6.** Absorption wavelengths (λ), oscillator strength (f) and molecular orbitals of **2** in water (CPCM) calculated by the wB97XD/6-311+G(2d,p)//wB97XD/6-311+G(2d,p) model, together with the experimental absorption and emission maxima.

| Transition | λ_th_/nm | f | MO | % | λ_exp_/nm |
| --- | --- | --- | --- | --- | --- |
| S_0_→T_1_ | 769 | 0.0000 | π_H_→π_L_* | 91 |  |
| S_0_→S_1_ | 526 | 0.9685 | π_H_→π_L_* | 92 | 438 |
| S_0_→T_2_ | 446 | 0.0000 | π_H-1_→π_L_* | 69 |  |
| S_0_→T_3_ | 403 | 0.0000 | π_H_→π_L+1_* | 50 |  |
| S_0_→T_4_ | 349 | 0.0000 | π_H-2_→π_L_* | 83 |  |
| S_0_→S_2_ | 335 | 0.0002 | π_H-2_→π_L_* | 86 |  |
| S_0_→S_3_ | 329 | 0.1406 | π_H_→π_L+1_* | 51 | 327 |
| S_0_→T_5_ | 325 | 0.0000 | π_H-3_→π_L_*  π_H_→π_L+8_* | 28  33 |  |
| S_0_→T_6_ | 312 | 0.0000 | π_H_→π_L+2_* | 48 |  |
| S_0_→S_4_ | 309 | 0.2076 | π_H-1_→π_L_*  π_H_→π_L+1_* | 49  30 |  |
| S_0_→T_7_ | 284 | 0.0000 | π_H-3_→π_L_*  π_H_→π_L+8_* | 32  23 |  |
| S_0_→T_8_ | 268 | 0.0000 | π_H-4_→π_L_*  π_H-4_→π_L+1_* | 29  33 |  |
| S_0_→S_5_ | 259 | 0.0124 | π_H_→π_L+2_* | 76 |  |
| S_0_→T_9_ | 246 | 0.0000 | π_H-3_→π_L+2_*  π_H_→π_L+3_* | 13  17 |  |
| S_0_→S_6_ | 243 | 0.0037 | π_H-3_→π_L_* | 40 |  |
| S_0_→T_10_ | 243 | 0.0000 | π_H-7_→π_L_*  π_H-3_→π_L+1_*  π_H_→π_L+1_* | 28  17  18 |  |
| S_0_→S_7_ | 235 | 0.1160 | π_H-3_→π_L_*  π_H_→π_L+8_* | 40  29 |  |
| S_0_→S_8_ | 229 | 0.4115 | π_H_→π_L+8_* | 44 |  |
| S_0_→S_9_ | 223 | 0.0081 | π_H_→π_L+4_* | 59 |  |
| S_0_→S_10_ | 218 | 0.0142 | π_H_→π_L+5_*  π_H_→π_L+6_* | 23  21 |  |
| S_1_→S_0_ | 581 | 0.9789 | π_H_→π_L_* | 95 | 640 |

**π_H−1_ π_H_**

**__**

**π_L_ π_L+1_**

**Figure S27.** Frontier molecular orbitals of **2**.

__

**Figure S28.** Effect of the S_0_→S_1_ transition on the electron density of **2**; increase and decrease of electron densities are represented by blue (+0.0001) and red (−0.0001), respectively.

**
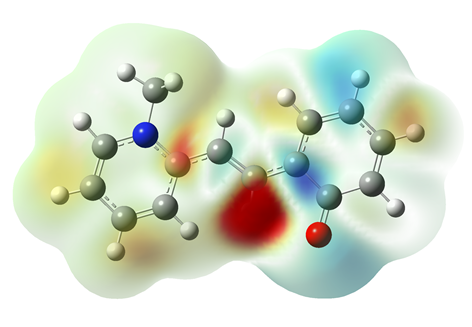
**

**Figure S29.** Effect of the S_1_→S_0_ transition on the electron density of **2**; increase and decrease of electron densities are represented by blue (+0.0001) and red (−0.0001), respectively.

**Table S7.** Absorption wavelengths (λ), oscillator strength (f) and molecular orbitals of **2^+^** in water (CPCM) calculated by the wB97XD/6-311+G(2d,p)//wB97XD/6-311+G(2d,p) model, together with the experimental absorption and emission maxima.

| Transition | λ_th_/nm | f | MO | % | λ_exp_/nm |
| --- | --- | --- | --- | --- | --- |
| S_0_→T_1_ | 508 | 0.0000 | π_H_→π_L_* | 64 |  |
| S_0_→S_1_ | 350 | 1.0748 | π_H_→π_L_* | 87 | 356 |
| S_0_→T_2_ | 339 | 0.0000 | π_H-2_→π_L_*  π_H-1_→π_L_*  π_H_→π_L+1_* | 26  27  22 |  |
| S_0_→T_3_ | 334 | 0.0000 | π_H-1_→π_L_*  π_H-1_→π_L+1_*  π_H-1_→π_L+2_* | 20  18  20 |  |
| S_0_→T_4_ | 324 | 0.0000 | π_H-2_→π_L+1_* | 27 |  |
| S_0_→T_5_ | 289 | 0.0000 | π_H-1_→π_L+3_*  π_H_→π_L+3_* | 20  24 |  |
| S_0_→S_2_ | 282 | 0.0922 | π_H-1_→π_L_* | 66 |  |
| S_0_→T_6_ | 272 | 0.0000 | π_H-3_→π_L_* | 36 |  |
| S_0_→T_7_ | 257 | 0.0000 | π_H-4_→π_L_* | 27 |  |
| S_0_→S_3_ | 256 | 0.0766 | π_H_→π_L+1_* | 54 |  |
| S_0_→T_8_ | 238 | 0.0000 | π_H-2_→π_L_*  π_H_→π_L+2_* | 24  25 |  |
| S_0_→T_9_ | 234 | 0.0000 | π_H-1_→π_L+3_*  π_H_→π_L+3_* | 39  26 |  |
| S_0_→S_4_ | 224 | 0.0758 | π_H-2_→π_L_* | 48 |  |
| S_0_→S_5_ | 221 | 0.0446 | π_H_→π_L+2_* | 53 |  |
| S_0_→T_10_ | 215 | 0.0000 | π_H-3_→π_L+1_* | 56 |  |
| S_0_→S_6_ | 211 | 0.0906 | π_H_→π_L+3_* | 42 |  |
| S_0_→S_7_ | 200 | 0.2331 | π_H-3_→π_L_* | 51 |  |
| S_0_→S_8_ | 196 | 0.2837 | π_H-1_→π_L+1_* | 38 |  |
| S_0_→S_9_ | 195 | 0.0479 | π_H_→π_L+4_* | 44 |  |
| S_0_→S_10_ | 194 | 0.4775 | π_H-1_→π_L+3_* | 52 |  |
| S_1_→S_0_ | 470 | 1.2300 | π_H_→π_L_* | 97 | 505 |

**π_H−1_ π_H_**

**__**

**π_L_ π_L+1_**

**Figure S30.** Frontier molecular orbitals of **2^+^**.

__

**Figure S31.** Effect of the S_0_→S_1_ transition on the electron density of **2^+^**; increase and decrease of electron densities are represented by blue (+0.0001) and red (−0.0001), respectively.


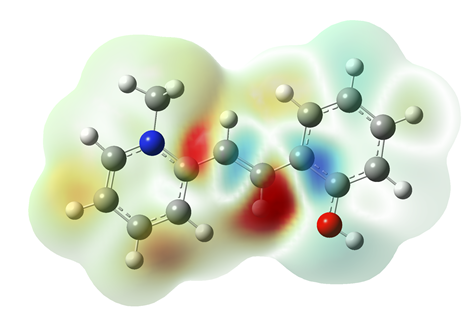


**Figure S32.** Effect of the S_1_→S_0_ transition on the electron density of **2^+^**; increase and decrease of electron densities are represented by blue (+0.0001) and red (−0.0001), respectively.

**Table S8.** Absorption wavelengths (λ), oscillator strength (f) and molecular orbitals of **3** in water (CPCM) calculated by the wB97XD/6-311+G(2d,p)//wB97XD/6-311+G(2d,p) model, together with the experimental absorption and emission maxima.

| Transition | λ_th_/nm | f | MO | % | λ_exp_/nm |
| --- | --- | --- | --- | --- | --- |
| S_0_→T_1_ | 766 | 0.0000 | π_H_→π_L_* | 89 |  |
| S_0_→S_1_ | 525 | 0.9095 | π_H_→π_L_* | 92 | 444 |
| S_0_→T_2_ | 444 | 0.0000 | π_H-1_→π_L_* | 67 |  |
| S_0_→T_3_ | 393 | 0.0000 | π_H_→π_L+1_* | 48 |  |
| S_0_→T_4_ | 345 | 0.0000 | π_H-2_→π_L_* | 82 |  |
| S_0_→T_5_ | 333 | 0.0000 | π_H_→π_L+7_* | 48 |  |
| S_0_→S_2_ | 331 | 0.0006 | π_H-2_→π_L_* | 85 |  |
| S_0_→S_3_ | 325 | 0.1533 | π_H_→π_L+1_* | 50 | 322 |
| S_0_→T_6_ | 316 | 0.0000 | π_H_→π_L+2_* | 44 |  |
| S_0_→S_4_ | 303 | 0.2142 | π_H-1_→π_L_*  π_H_→π_L+1_* | 48  30 |  |
| S_0_→T_7_ | 288 | 0.0000 | π_H-3_→π_L_*  π_H_→π_L+7_* | 30  19 |  |
| S_0_→T_8_ | 270 | 0.0000 | π_H-6_→π_L_*  π_H-6_→π_L+1_* | 38  35 |  |
| S_0_→S_5_ | 264 | 0.0374 | π_H_→π_L+2_* | 71 |  |
| S_0_→T_9_ | 248 | 0.0000 | π_H-7_→π_L_*  π_H-3_→π_L+2_* | 16  16 |  |
| S_0_→T_10_ | 247 | 0.0000 | π_H_→π_L+6_* | 40 |  |
| S_0_→S_6_ | 243 | 0.0020 | π_H_→π_L+3_* | 48 |  |
| S_0_→S_7_ | 238 | 0.3756 | π_H_→π_L+7_* | 59 |  |
| S_0_→S_8_ | 232 | 0.0449 | π_H_→π_L+6_*  π_H_→π_L+9_* | 28  18 |  |
| S_0_→S_9_ | 231 | 0.1786 | π_H-4_→π_L_*  π_H-1_→π_L+1_* | 27  20 |  |
| S_0_→S_10_ | 219 | 0.0002 | π_H_→π_L+4_* | 39 |  |
| S_1_→S_0_ | 592 | 0.9405 | π_H_→π_L_* | 95 | 649 |

**π_H−1_ π_H_**

**__**

**π_L_ π_L+1_**

**Figure S33.** Frontier molecular orbitals of **3**.


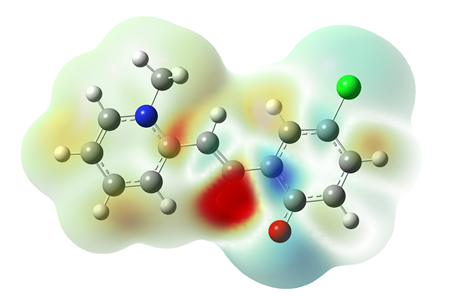


Figure S34. Effect of the S_1_→S_0_ transition on the electron density of 3; increase and decrease of electron densities are represented by blue (+0.0001) and red (−0.0001), respectively.

**Table S9.** Absorption wavelengths (λ), oscillator strength (f) and molecular orbitals of **3^+^** in water (CPCM) calculated by the wB97XD/6-311+G(2d,p)//wB97XD/6-311+G(2d,p) model, together with the experimental absorption and emission maxima.

| Transition | λ_th_/nm | f | MO | % | λ_exp_/nm |
| --- | --- | --- | --- | --- | --- |
| S_0_→T_1_ | 501 | 0.0000 | π_H_→π_L_* | 53 |  |
| S_0_→T_2_ | 347 | 0.0000 | π_H-1_→π_L_* | 41 |  |
| S_0_→S_1_ | 346 | 0.9846 | π_H_→π_L_* | 81 | 360 |
| S_0_→T_3_ | 338 | 0.0000 | π_H-2_→π_L_*  π_H_→π_L+3_* | 26  22 |  |
| S_0_→T_4_ | 324 | 0.0000 | π_H-2_→π_L_* | 28 |  |
| S_0_→T_5_ | 295 | 0.0000 | π_H_→π_L+3_* | 25 |  |
| S_0_→S_2_ | 285 | 0.1676 | π_H-1_→π_L_* | 67 | 324 |
| S_0_→T_6_ | 273 | 0.0000 | π_H-5_→π_L_* | 34 |  |
| S_0_→T_7_ | 259 | 0.0000 | π_H-6_→π_L_*  π_H-4_→π_L_* | 18  21 |  |
| S_0_→S_3_ | 252 | 0.0782 | π_H_→π_L+1_* | 41 |  |
| S_0_→T_8_ | 237 | 0.0000 | π_H-2_→π_L_*  π_H_→π_L+2_* | 16  22 |  |
| S_0_→T_9_ | 233 | 0.0000 | π_H-1_→π_L+3_* | 47 |  |
| S_0_→S_4_ | 228 | 0.1923 | π_H_→π_L+2_*  π_H_→π_L+3_* | 28  28 |  |
| S_0_→S_5_ | 220 | 0.0336 | π_H-2_→π_L_* | 40 |  |
| S_0_→T_10_ | 216 | 0.0000 | π_H-5_→π_L+1_* | 41 |  |
| S_0_→S_6_ | 215 | 0.1906 | π_H_→π_L+3_* | 45 |  |
| S_0_→S_7_ | 201 | 0.2850 | π_H-5_→π_L_* | 46 |  |
| S_0_→S_8_ | 199 | 0.0213 | π_H_→π_L+7_* | 28 |  |
| S_0_→S_9_ | 198 | 0.2303 | π_H-1_→π_L+1_*  π_H-1_→π_L+2_* | 24  21 |  |
| S_0_→S_10_ | 195 | 0.0164 | π_H_→π_L+4_* | 45 |  |
| S_1_→S_0_ | 472 | 1.1798 | π_H_→π_L_* | 96 | 524 |


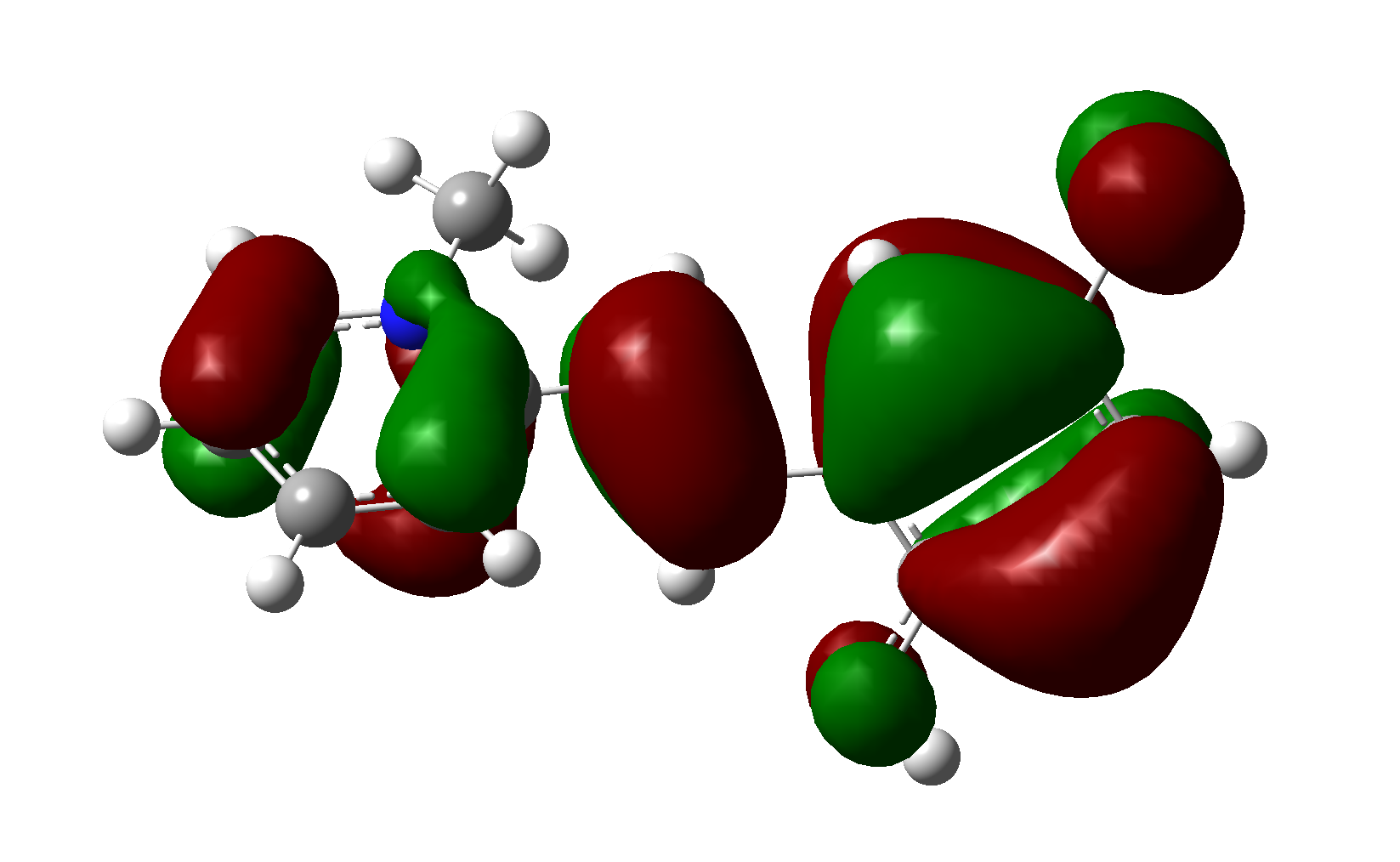

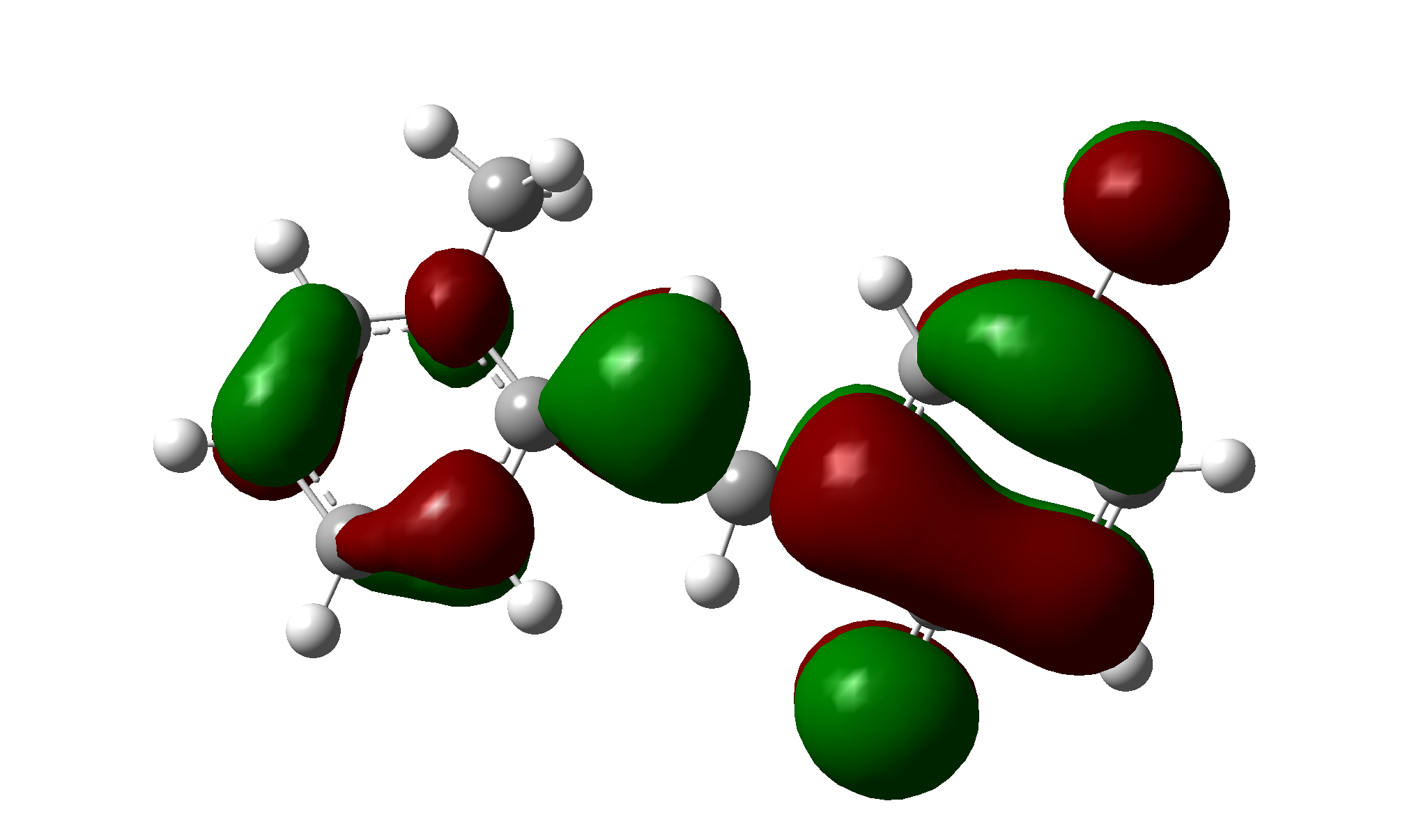


**π_H−1_ π_H_**

**__**

**π_L_ π_L+1_**

**Figure S35.** Frontier molecular orbitals of **3^+^**.

**
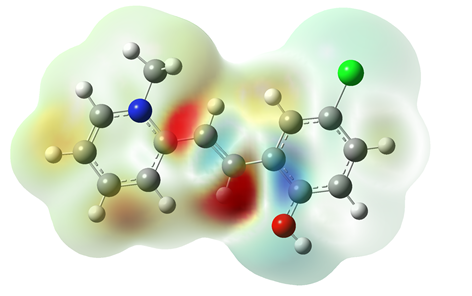
**

**Figure S36.** Effect of the S_1_→S_0_ transition on the electron density of **3^+^**; increase and decrease of electron densities are represented by blue (+0.0001) and red (−0.0001), respectively.

**2** pH 11 **2** pH 4 **2** H_0_ −3

**Figure S37.** Femtosecond-transient absorption (fs-TA) measurements of compound **2** in buffered water at pH 11 (left), pH 4 (middle), and H_0_ −3 (right) (λ_exc_ = 400 nm): panel A, experimental 3D matrix reporting color-coded ΔA as a function of wavelength and time (ΔA > 0 green-yellow, ΔA < 0 blue); panel B, representative spectra at different delay times and representative kinetics (inset) at different wavelengths; panel C, EAS (evolution-associated spectra) obtained by Global Analysis.

**3** pH 4 (CaF_2_)

**Figure S38.** Femtosecond-transient absorption (fs-TA) measurements of compound **3** in buffered water at pH 4 (λ_exc_ = 400 nm, white-light probe generated by CaF_2_ crystal): panel A, experimental 3D matrix reporting color-coded ΔA as a function of wavelength and time (ΔA > 0 green-yellow-orange, ΔA < 0 blue); panel B, representative spectra at different delay times and representative kinetics (inset) at different wavelengths; panel C, EAS (evolution-associated spectra) obtained by Global Analysis, together with the steady-state absorption spectrum of deprotonated/zwitterionic **3** (purple-shaded area).

Table S10. Rate constants for compounds 1-3 in buffered water at H_0_ −3, pH 4, and pH 11: fluorescence quantum yields (Φ_F_), lifetimes of S_1_ as obtained by femtosecond TA (τ_S1_), fluorescence rate constants (k_F_ = Φ_F_/τ_S1_), non-radiative rate constants (k_nr_ = (1−Φ_F_)/τ_S1_), ESPT efficiencies (Φ_ESPT_), times for the ESPT process as obtained by femtosecond TA (τ_ESPT_), and ESPT rate constants (k_ESPT_ = Φ_ESPT_/τ_ESPT_).

| **Compound** | **pH/H_0_** | **Φ_F_** | **τ_S1_ / ps** | **k_F_ / s^−1^** | **k_nr_ / s^−1^** | **Φ_ESPT_** | **τ_ESPT_ / ps** | **k_ESPT_ / s^−1^** |
| --- | --- | --- | --- | --- | --- | --- | --- | --- |
| **1^+^** | −3 | 0.0014 | 6.6 | 2.1×10^8^ | 1.5×10^11^ | **-** |  |  |
| **1^+^** | 4 | 0.0013 | 4.2 | 3.1×10^8^ | 2.3×10^11^ | **-** |  |  |
| **1** | 11 | 0.00030 | 1.4 | 2.1×10^8^ | 7.1×10^11^ | **-** |  |  |
| **2^+^** | −3 | 0.0050 | 15 | 3.3×10^8^ | 0.66×10^11^ | **-** |  |  |
| **2^+^** | 4 | 0.0011 | 10 | 1.1×10^8^ | 1.0×10^11^ | 0.88 | 2.6 | 3.38×10^11^ |
| **2** | 11 | 0.00056 | 5.6 | 1.0×10^8^ | 1.8×10^11^ | - |  |  |
| **3^+^** | −3 | 0.013 | 48 | 2.7×10^8^ | 0.21×10^11^ | - |  |  |
| **3^+^** | 4 | 0.00088 | 13 | 0.68×10^8^ | 0.77×10^11^ | 0.98 | 3.5 | 2.80×10^11^ |
| **3** | 11 | 0.00065 | 9.2 | 0.71×10^8^ | 1.1×10^11^ | **-** |  |  |

**1** pH 11 **1** pH 4

**Figure S39.** Femtosecond fluorescence up-conversion (UC) measurements of compound **1** in buffered water at pH 11 (left) and pH 4 (right) (λ_exc_ = 400 nm): panel A, experimental 3D matrix reporting color-coded fluorescence intensity as a function of wavelength and time (I_F_ increases following the scale green-yellow-orange-red); panel B, representative spectra at different delay times and representative kinetics (inset) at different wavelengths; panel C, EAS (evolution-associated spectra) obtained by Global Analysis.

**2** pH 11 **2** pH 4

**Figure S40.** Femtosecond fluorescence up-conversion (UC) measurements of compound **2** in buffered water at pH 11 (left) and pH 4 (right) (λ_exc_ = 400 nm): panel A, experimental 3D matrix reporting color-coded fluorescence intensity as a function of wavelength and time (I_F_ increases following the scale green-yellow-orange-red); panel B, representative spectra at different delay times and representative kinetics (inset) at different wavelengths; panel C, EAS (evolution-associated spectra) obtained by Global Analysis.

**3** pH 11 **3** pH 4

**Figure S41.** Femtosecond fluorescence up-conversion (UC) measurements of compound **3** in buffered water at pH 11 (left) and pH 4 (right) (λ_exc_ = 400 nm): panel A, experimental 3D matrix reporting color-coded fluorescence intensity as a function of wavelength and time (I_F_ increases following the scale green-yellow-orange-red); panel B, representative spectra at different delay times and representative kinetics (inset) at different wavelengths; panel C, EAS (evolution-associated spectra) obtained by Global Analysis.

**Table S11.** Femtosecond transient absorption **(**TA) and fluorescence up-conversion (UC) results of compounds **1-3** in buffered water at pH 11, pH 4, and H_0_ −3 obtained by Global Analysis and their assignment.

| **Compound** | **pH 11** | | **pH 4** | | | **H_0_ −3** | | **Assignment** |
| --- | --- | --- | --- | --- | --- | --- | --- | --- |
|  | **τ_TA_ /ps** | **τ_UC_ /ps** | **τ_TA_ /ps** | | **τ_UC_ /ps** | **τ_TA_ /ps** | **τ_UC_ /ps** |  |
|  |  |  | **Ti:sapphire** | **CaF_2_** |  |  |  |  |
| **1** | 0.60 | 0.60 | 0.58 |  |  | 0.58 | 0.58 | Solv. |
|  |  |  | 4.2 |  | 3.7 | 6.6 | 5.9 | **S_1_ ACID** |
|  | 1.4 | 1.9 |  |  |  |  |  | **S_1_ BASE** |
| **2** | 0.33 |  |  |  |  | 1.2 |  | Solv. |
|  | 0.85 | 0.85 | 0.83 |  | 0.49 | 15 |  | Solv.**/S_1_ ACID** |
|  |  |  | 2.6 |  | 3.2 |  |  | **ESPT** |
|  | 5.6 | 5.2 | 10 |  | 11 |  |  | **S_1_ BASE** |
|  |  |  | 53 |  |  |  |  | **S_0,hot_ BASE** |
|  |  |  | rest |  |  |  |  | **S_0_ BASE** |
| **3** | 0.47 | 0.47 |  |  |  | 1.6 | 2.8 | Solv. |
|  | 0.84 | 0.74 | 0.68 | 0.68 | 0.51 | 48 | 61 | Solv./**S_1_ ACID** |
|  |  |  | 3.5 | 5.5 | 3.4 |  |  | **ESPT** |
|  | 9.2 | 9.2 | 13 | 9 | 13 |  |  | **S_1_ BASE** |
|  |  |  | 62 |  |  |  |  | **S_0,hot_ BASE** |
|  |  |  | rest | rest |  |  |  | **S_0_ BASE** |

TRANES **2** pH 4

**Figure S42.** Time-resolved area-normalized emission spectra (TRANES) analysis of femtosecond fluorescence up-conversion (UC) data of compound **2** in buffered water at pH 4 (λ_exc_ = 400 nm): panel A, 3D matrix reporting color-coded TRANES intensity as a function of wavelength and time; panel B, intensity variations of TRANES as a function of time at significant wavelengths; panel C, concentration profiles for the transient species detected by Global Analysis; panels D and E, TRANES evolution over time calculated in proper delay time intervals together with the EAS obtained by Global Analysis (dashed lines) reported as limit spectra.

**Figure S43.** Nanosecond laser flash photolysis measurements of compound **1** in buffered water at pH 4 (λ_exc_ = 355 nm): kinetic recorded at λ = 450 nm.

**Figure S44.** Nanosecond laser flash photolysis measurements of compound **2** in buffered water at pH 4 (λ_exc_ = 355 nm): kinetics recorded at the maximum wavelength of the transient band (λ = 450 nm) and its fit in areated (panel A) and deareated (panel B) solutions.

**Figure S45.** Nanosecond laser flash photolysis measurements of compound **3** in buffered water at pH 4 (λ_exc_ = 355 nm): kinetic recorded at the maximum wavelength of the transient band (λ = 450 nm) and its fit in areated solution.

**Figure S46.** Nanosecond laser flash photolysis measurements of compound **3** in buffered water at pH 6 in the presecne of acetate (λ_exc_ = 355 nm): panel A, representative spectra at different delay times; panel B, kinetic recorded at the maximum wavelength of the transient band (λ = 450 nm) and its fit in deareated solution; panel C, experimental 3D matrix reporting color-coded ΔOD as a function of wavelength and time (ΔOD > 0 yellow-red, ΔOD < 0 blue); panel D, scheme representing the proton scavenger activity of acetate.

***References***

[52] C. Bonomo, P. G. Bonacci, D. A. Bivona, A. Mirabile, D. Bongiorno, E. Nicitra, A. Marino, C. Bonaccorso, G. Consiglio, C. G. Fortuna, S. Stefani, N. Musso, *Antibiotics* **2023**, *12*, 1308.

[53] M. Montalti, A. Credi, L. Prodi, M. T. Gandolfi, *Handbook of Photochemistry*, CRC Press, **2006**.

[54] Th. Förster, *Naturwissenschaften* **1949**, *36*, 186–187.

[55] Th. Förster, *Zeitschrift für Elektrochemie und angewandte physikalische Chemie* **1950**, *54*, 531–535.

[56] A. Weller, *Zeitschrift für Elektrochemie, Berichte der Bunsengesellschaft für physikalische Chemie* **1954**, *58*, 849–853.

[57] W. Urban, A. Weller, *Berichte der Bunsengesellschaft für physikalische Chemie* **1963**, *67*, 787–791.

[58] A. Weller, *Discuss. Faraday Soc.* **1959**, *27*, 28–33.

[59] A. Weller, *Prog. React. Kinet* **1961**, *1*, 187–214.

[60] T. Bianconi, A. Cesaretti, P. Mancini, N. Montegiove, E. Calzoni, A. Ekbote, R. Misra, B. Carlotti, *J. Phys. Chem. B* **2023**, *127*, 1385–1398.

[61] A. Cesaretti, T. Bianconi, M. Coccimiglio, N. Montegiove, Y. Rout, P. L. Gentili, R. Misra, B. Carlotti, *J. Phys. Chem. C* **2022**, *126*, 10429–10440.

[62] J. J. Snellenburg, S. Laptenok, R. Seger, K. M. Mullen, I. H. M. van Stokkum, *Journal of Statistical Software* **2012**, *49*, 1–22.

[63] F. M, *Inc, Wallingford CT* **2009**, *201*.

[64] T. Yanai, D. P. Tew, N. C. Handy, *Chemical Physics Letters* **2004**, *393*, 51–57.

[65] T. Yanai, R. J. Harrison, N. C. Handy, *Molecular Physics* **2005**, *103*, 413–424.

[66] V. Barone, M. Cossi, *J. Phys. Chem. A* **1998**, *102*, 1995–2001.
